# B cells mediate lung ischemia/reperfusion injury by recruiting classical monocytes via synergistic B cell receptor/TLR4 signaling

**DOI:** 10.1172/JCI170118

**Published:** 2024-01-23

**Authors:** Khashayar Farahnak, Yun Zhu Bai, Yuhei Yokoyama, Deniz B. Morkan, Zhiyi Liu, Junedh M. Amrute, Alejandro De Filippis Falcon, Yuriko Terada, Fuyi Liao, Wenjun Li, Hailey M. Shepherd, Ramsey R. Hachem, Varun Puri, Kory J. Lavine, Andrew E. Gelman, Ankit Bharat, Daniel Kreisel, Ruben G. Nava

**Affiliations:** 1 Department of Surgery,; 2 Department of Medicine, and; 3 Department of Pathology and Immunology, Washington University School of Medicine, Saint Louis, Missouri, USA.; 4 Department of Surgery, Northwestern University, Chicago, Illinois, USA.

**Keywords:** Immunology, Transplantation, Chemokines, Monocytes, Organ transplantation

## Abstract

Ischemia/reperfusion injury–mediated (IRI-mediated) primary graft dysfunction (PGD) adversely affects both short- and long-term outcomes after lung transplantation, a procedure that remains the only treatment option for patients suffering from end-stage respiratory failure. While B cells are known to regulate adaptive immune responses, their role in lung IRI is not well understood. Here, we demonstrated by intravital imaging that B cells are rapidly recruited to injured lungs, where they extravasate into the parenchyma. Using hilar clamping and transplant models, we observed that lung-infiltrating B cells produce the monocyte chemokine CCL7 in a TLR4-TRIF–dependent fashion, a critical step contributing to classical monocyte (CM) recruitment and subsequent neutrophil extravasation, resulting in worse lung function. We found that synergistic BCR-TLR4 activation on B cells is required for the recruitment of CMs to the injured lung. Finally, we corroborated our findings in reperfused human lungs, in which we observed a correlation between B cell infiltration and CM recruitment after transplantation. This study describes a role for B cells as critical orchestrators of lung IRI. As B cells can be depleted with currently available agents, our study provides a rationale for clinical trials investigating B cell–targeting therapies.

## Abstract 2

Synergistic B cell receptor/TLR4 signaling on lung-infiltrating B cells is critical for orchestrating lung ischemia reperfusion injury, by facilitating the recruitment of CCR2+ classical monocytes.

## Graphical abstract

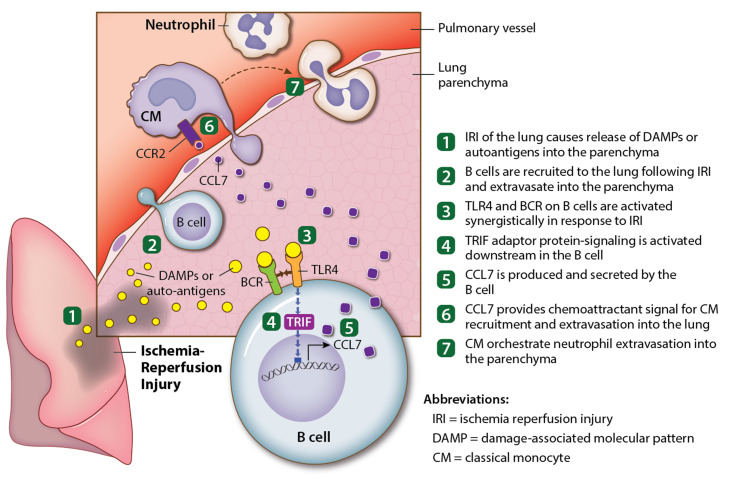

## Introduction

Primary graft dysfunction (PGD) is a common early complication after lung transplantation that adversely affects both short- and long-term outcomes. This includes both early morbidity and mortality as well as a higher risk for late graft loss due to chronic rejection. Our center has reported that the incidence of severe PGD has not changed over time (1, 2). Ischemia/reperfusion injury (IRI) is widely considered a major initiating event causing PGD. We and others have shown that neutrophils are critical mediators of lung IRI (3–5). Our group has described several important steps leading to their recruitment and extravasation after murine lung transplantation-induced IRI. For example, using intravital 2-photon microscopy, we observed that monocytes mediate neutrophil extravasation in lung grafts during IRI (6). More recently, we reported that graft-resident donor nonclassical monocytes (NCMs) and spleen-derived recipient CCR2^+^ classical monocytes (CMs) orchestrate the recruitment and extravasation of neutrophils, respectively (7–9). We have also shown that extended cold ischemic graft storage enhances granulocyte colony-stimulating factor–mediated (G-CSF–mediated) granulopoiesis and neutrophil graft infiltration, resulting in exacerbation of IRI and potentiation of alloimmunity (4, 10).

B cells can regulate immune responses through antibody production, antigen presentation, and cytokine secretion (11). In addition to their capacity to enhance inflammatory responses, B cells are also capable of dampening inflammation (12). In lung transplantation, B cells are known mediators of antibody-mediated rejection (13) and are targeted in patients who suffer from this complication (14). The expression of TLRs on B cells provides a cell-intrinsic mechanism by which innate signals can regulate adaptive immune responses (15). It is known that the dual expression of and interaction between TLRs and the B cell receptor (BCR) play a crucial role not only in the B cell response to pathogen challenge, but also for B cell survival, development, and antibody production under both physiological and pathological conditions. In vitro studies have demonstrated that synergistic BCR/TLR signaling on B cells leads to a diversified antibody response secondary to somatic hypermutation (16) and class-switch DNA recombination (CSR) (17, 18). Several studies have suggested that B cells contribute to IRI and may regulate responses of innate immune cells that we know play a critical role in mediating PGD, such as monocytes and neutrophils (19–21). However, whether B cells play a role in lung IRI has not been fully elucidated. Additionally, approaches that target immune cells to prevent or attenuate PGD after lung transplantation have not been implemented in the clinical setting. Therefore, a better understanding and characterization of the immune response and the mechanisms by which IRI contributes to tissue damage are paramount for clinical translation into improved outcomes.

In this study, we demonstrated that peripheral B cells enter the lung and orchestrate the recruitment of CCR2^+^ CMs into the lung graft. We observed that TLR4 is activated on lung-infiltrating B cells shortly after lung reperfusion, promoting the production of the monocyte chemokine CCL7 in a TRIF-dependent fashion. This step plays a critical role in driving CM recruitment and subsequent neutrophil extravasation, resulting in worse lung function. We show that TLR4 stimulation was synergistic with BCR activation and the lack of BCR engagement precluded chemotaxis of CMs to the lung. These data support a significant and deleterious role of B cells in lung IRI. As clinically successful B cell depletion strategies have been developed, our findings provide an impetus for investigating future specific B cell–targeting therapies for lung IRI and transplantation.

## Results

### B cells are recruited to the lung after IRI in a murine hilar clamping model.

**Figure 1:**
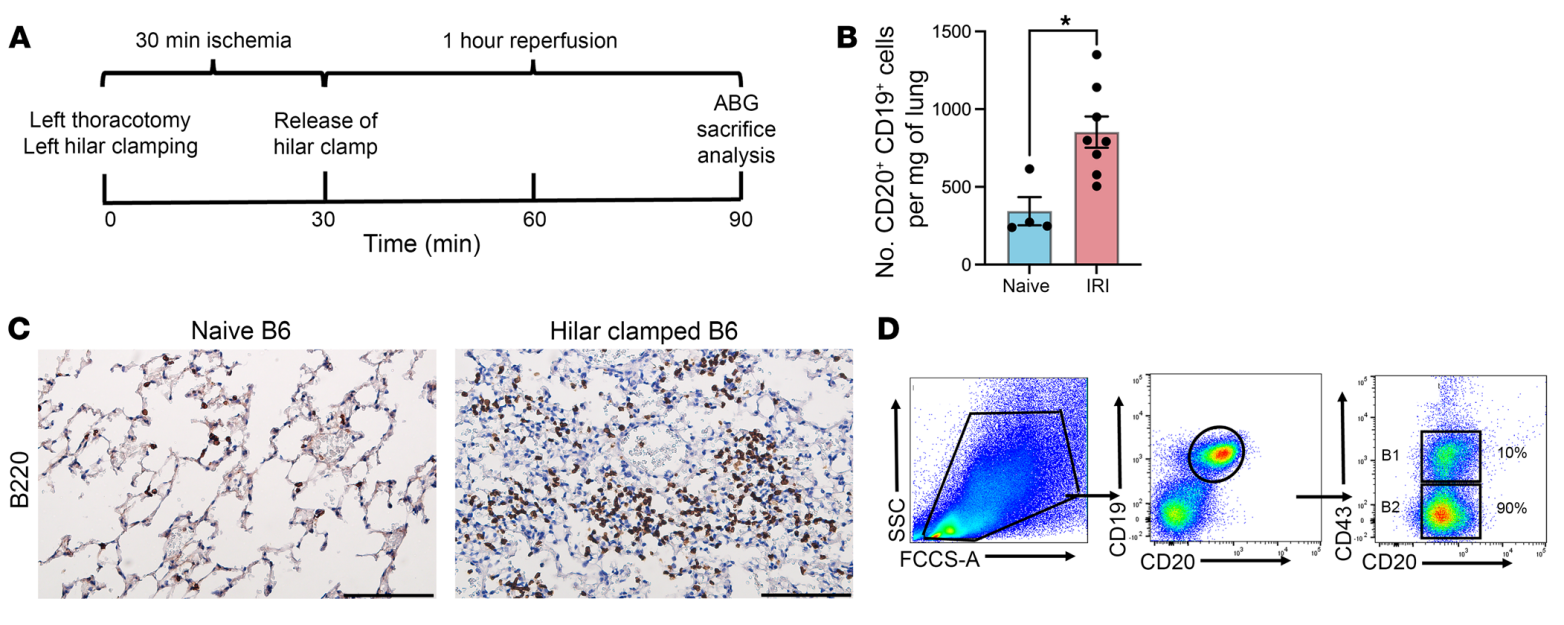
B cell recruitment to the lung increases after ischemia/reperfusion injury. (**A**) Mice underwent left pulmonary clamping with 30 minutes of ischemia and 60 minutes of reperfusion. The left lung was harvested for analysis. (**B**) Flow cytometric analysis of total number of CD19^+^CD20^+^ B cells per milligram (mg) of lung tissue in IRI compared with naive mice. (**C**) Representative IHC staining of lung tissue for B220 expression in IRI. Original magnification, ×40; scale bars: 100 μm. (**D**) Gating strategy for murine B2 B cells (CD19^+^CD20^+^CD43^–^) and B1 B cells (CD19^+^CD20^+^CD43^+^). B1 and B2 B cells are gated on live (forward vs. side scatter or FCCS-A vs. SCC) CD45^+^ cells. Results are presented as mean ± SEM. *n* = 4–8. *P* values were calculated by Mann-Whitney test. *P < 0.05.

Tissue damage after IRI is determined by the extent and duration of ischemia and the downstream deleterious effects of organ reperfusion. The combination of increased vascular permeability and increased cellular signaling amplifies the recruitment and infiltration of circulating leukocytes into the lung (22, 23). To explore the role of B cells in lung IRI, we used a murine left pulmonary hilar clamping model, where 30 minutes of warm ischemia is followed by 60 minutes of reperfusion (Figure 1A). Using flow cytometric analysis and IHC staining, we observed that the total number of B cells (CD20^+^CD19^+^) significantly increased in the injured lung 1 hour after reperfusion in C57BL/6 (B6) WT mice subjected to left hilar clamping, when compared with naive B6 WT mice (Figure 1, B and C). B cells are divided into innate B1 and conventional B2 B cells, which have distinct origins, phenotypes, functions, and anatomical localization (24). In mice, B2 B cells are defined as CD20^+^CD19^+^CD43^–^ and B1 B cells are defined as CD20^+^CD19^+^CD43^+^. We found that B1 B cells account for only 4%–10% of total lung B cells, while the majority are B2 B cells (Figure 1D).

### B cells worsen lung function after IRI by increasing neutrophil extravasation.

**Figure 2:**
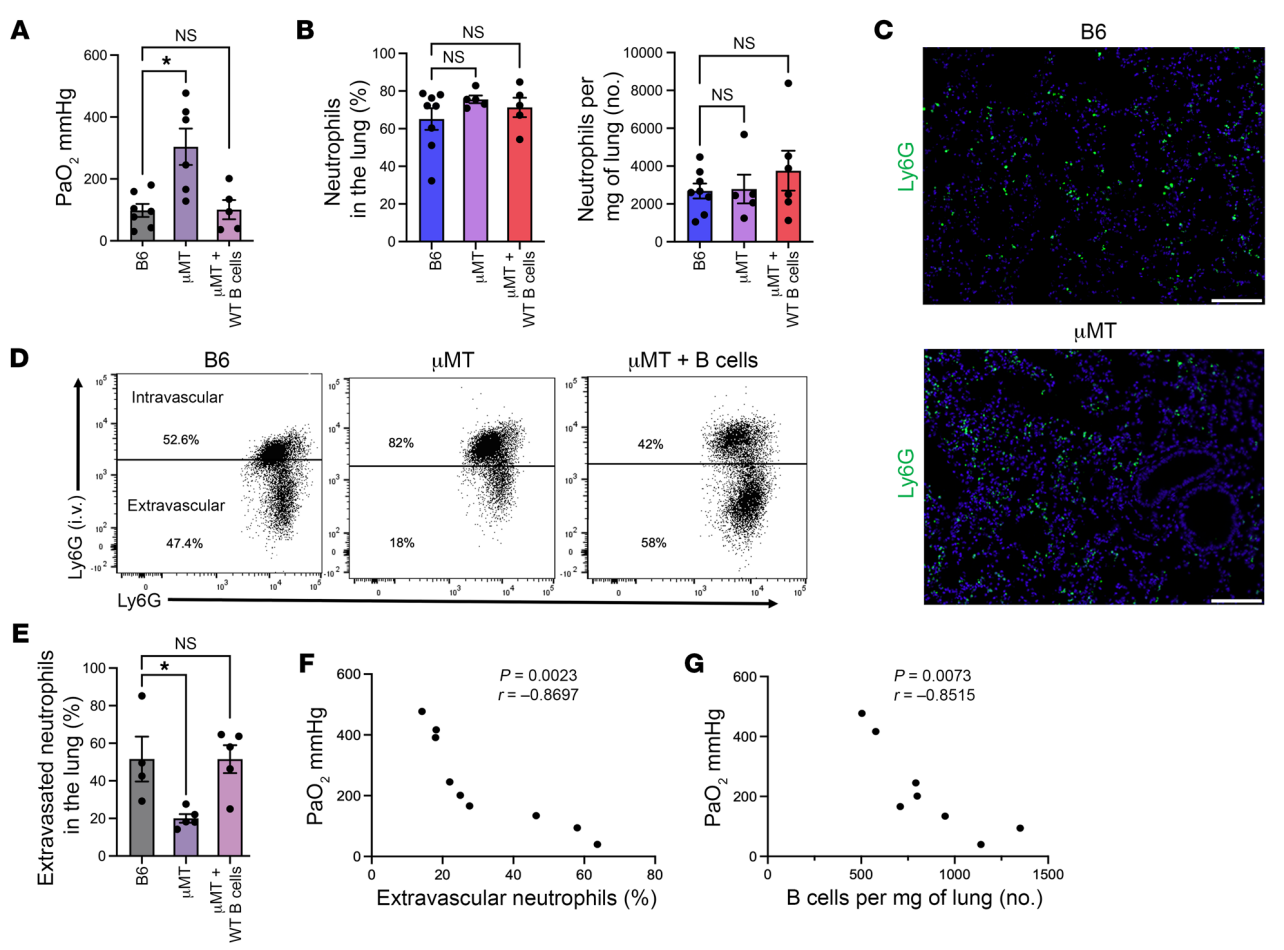
B cells increase neutrophil extravasation after ischemia/reperfusion injury, leading to worse lung function. After 1 hour of reperfusion, left lung function was assessed by ABG measurement immediately prior to sacrifice. Flow cytometry was used to analyze myeloid populations. (**A**) PaO_2_ values after IRI are significantly improved in mice lacking B cells (μMT) compared with those in B6 WT mice. Adoptive transfer of approximately 10^6^ splenic B cells from B6 WT mice into μMT mice abrogates this improvement in oxygenation. (**B**) Percentage of and total number per milligram of lung tissue of neutrophils (CD45^+^Ly6G^+^Ly6C^+^) recruited to the lung after IRI. (**C**) Representative immunofluorescence staining of lung tissue for Ly6G expression after IRI. Original magnification, ×20; scale bars: 200 μm. (**D**) Representative dot plots of intravascular versus extravascular neutrophils in the lungs. Neutrophil extravasation was determined with flow cytometry by injecting fluorochrome-labeled neutrophil-specific anti-Ly6G antibodies i.v. 5 minutes prior to sacrifice. (**E**) Quantification of extravasated neutrophils in the lung after IRI. (**F**) Negative correlation between PaO_2_ values and percentage of extravascular neutrophils after IRI. (**G**) Negative correlation between PaO_2_ values and number of B cells after IRI. Pearson’s correlation coefficients (*r*) were significant (**F** and **G**). Results are presented as mean ± SEM. *n* = 4–9. *P* values were calculated by Kruskal-Wallis test. *P < 0.05 (**A**, **B**, and **E**).

To determine whether B cells play a functional role in lung IRI, we subjected B6 WT and B cell–deficient (μMT) mice to left pulmonary hilar clamping. Of note, μMT mice have a small number of B1 B cells in lung and spleen at baseline (Supplemental Figure 1, A and B; supplemental material available online with this article; https://doi.org/10.1172/JCI170118DS1) but completely lack B2 B cells. Arterial blood gases (ABGs) were obtained to assess lung function, as previously described (25), and the left lung was harvested for flow cytometric analysis. We observed that oxygenation was significantly improved in μMT mice. This improvement in oxygenation was abrogated when B cells from the spleen of B6 WT mice were injected i.v. into μMT mice 24 hours before left hilar clamping (Figure 2A). Tissue damage during lung IRI is dependent on neutrophil recruitment and their subsequent extravasation (5, 7–9). Therefore, we next set out to determine whether neutrophil recruitment and extravasation was altered by B cells. Neutrophil extravasation was determined flow cytometrically by injecting fluorochrome-labeled neutrophil-specific anti-Ly6G antibodies i.v. 5 minutes before lung harvest, as previously described (7). Interestingly, neutrophil recruitment to the injured lungs was comparable irrespective of whether B cells were present, demonstrated both by flow cytometry and immunofluorescence staining of Ly6G (Figure 2, B and C). However, we observed a significantly decreased proportion of extravasated neutrophils in μMT mice versus WT mice. Adoptive transfer of splenic WT B cells into μMT mice increased the percentage of extravasated neutrophils to levels comparable to WT mice (Figure 2, D and E). Oxygenation was inversely correlated with the percentage of extravasated neutrophils (Figure 2F) and the number of lung-infiltrating B cells (Figure 2G). Taken together, these findings demonstrate that B2 B cells contribute to lung IRI by increasing neutrophil extravasation.

### Improvement of lung function in the absence of B cells is associated with a significant reduction in the abundance and extravasation of CCR2^+^CMs in the lung.

**Figure 3:**
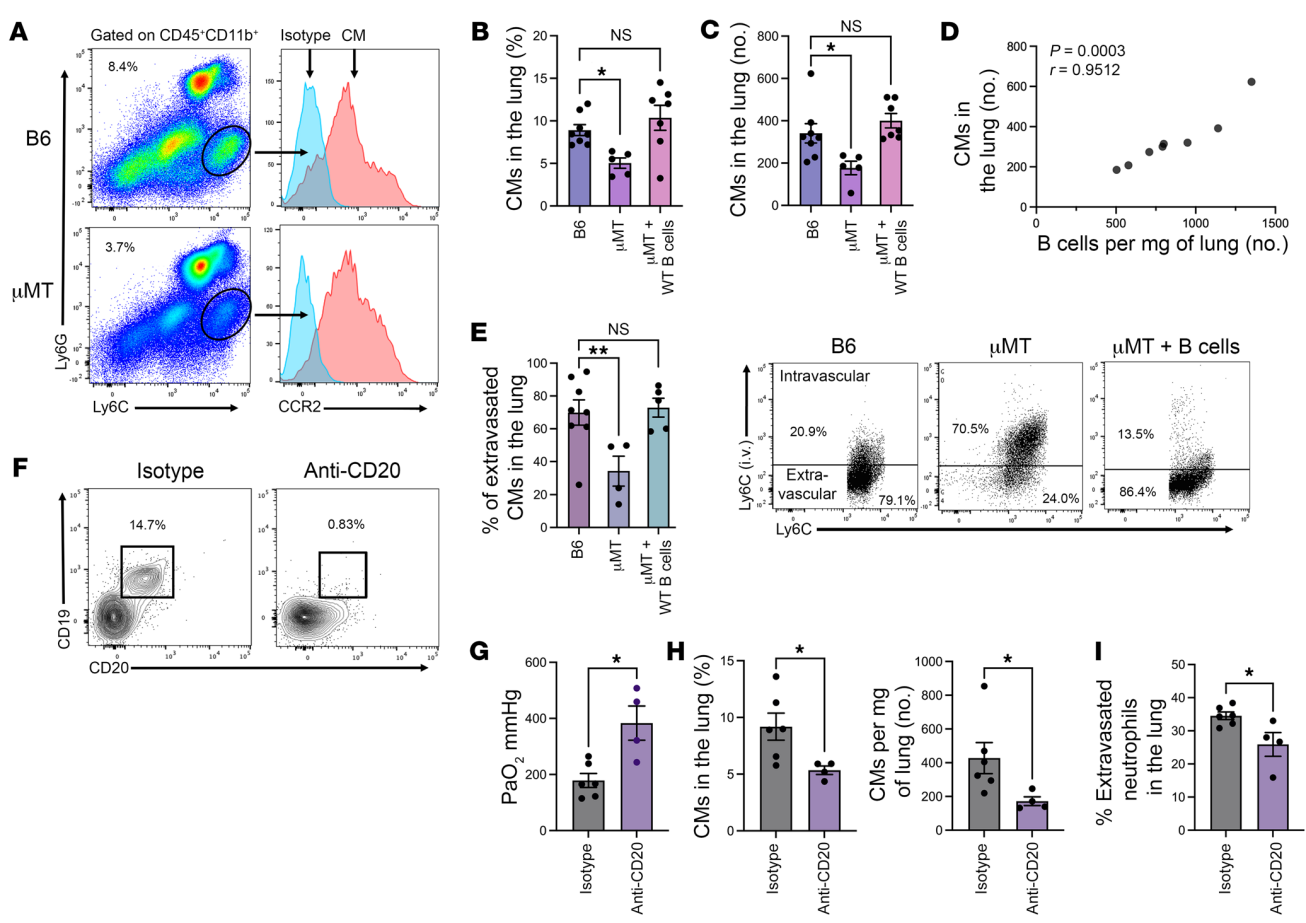
Lung function improvement in the absence of B cells is associated with a significant reduction in the abundance of CCR2^+^ classical monocytes in the lung. (**A**) Flow cytometry gating strategy for classical monocytes (CD45^+^CD11b^+^Ly6G^–^Ly6C^hi^CCR2^+^) between hilar clamped B6 WT mice and μMT mice. (**B**) Percentage of and (**C**) total number per milligram of lung tissue of classical monocytes recruited to the lung after IRI in B6 WT mice and μMT mice with and without adoptive transfer of B6 WT B cells. (**D**) Positive correlation between number of B cells and number of classical monocytes in the lung after IRI. Pearson’s correlation coefficient (*r*) was significant. (**E**) Quantification and representative flow cytometry dot plots of extravascular classical monocytes in the lungs after adoptive transfer of B6 WT B cells, which are significantly lower in μMT mice compared with B6 WT and μMT mice with WT B cells. Lung recipients were treated with anti-CD20–specific or isotype control antibodies and analyzed for (**F**) intragraft B cell depletion, (**G**) lung function (PaO_2_), (**H**) the relative proportion and total number of classical monocytes, and (**I**) neutrophil extravasation. *n* = 4–8. Data shown in **E** and **F** are representative dot and contour plots where *n* > 4 per group. Results are presented as mean ± SEM. (**B**, **C**, and **E**) *P* values were calculated by Kruskal-Wallis test. *P < 0.05, **P < 0.01. (**G**–**I**) *P* values were calculated by Mann-Whitney test. *P < 0.05.

Our group has recently reported that recipient spleen-derived CCR2^+^ CMs mediate neutrophil extravasation in lung grafts during IRI through their production of IL-1β (7). Therefore, we set out to investigate whether CM recruitment and extravasation to the lung is affected by the presence of B cells after IRI. CM extravasation was determined with i.v. injection of fluorochrome-labeled monocyte-specific anti-Ly6C antibodies, as previously carried out for neutrophil extravasation experiments. CMs were defined by flow cytometry as CD45^+^CD11b^+^Ly6G^–^Ly6C^hi^CCR2^+^ cells (Figure 3A). Indeed, we found that percentages and abundance of CMs in the lung were significantly reduced in μMT mice compared with WT mice. Adoptive transfer of splenic WT B cells into μMT mice increased percentages and abundance of CMs to levels comparable to those observed in WT mice (Figure 3, B and C). In addition, we noted a positive correlation between the number of B cells and CMs in the injured lung (Figure 3D). We also observed a significantly increased proportion of extravascular CMs in the presence of B cells (Figure 3E). Finally, B cell depletion using anti-CD20 antibodies prior to hilar clamping improved lung function (Figure 3, F and G) and was associated with decreases in the percentage and number of CMs in the lung and in neutrophil extravasation (Figure 3, H and I). Taken together, these data suggest that B cells contribute to the recruitment and extravasation of CMs to the lung, which facilitates neutrophil extravasation and promotes injury.

**Figure 4:**
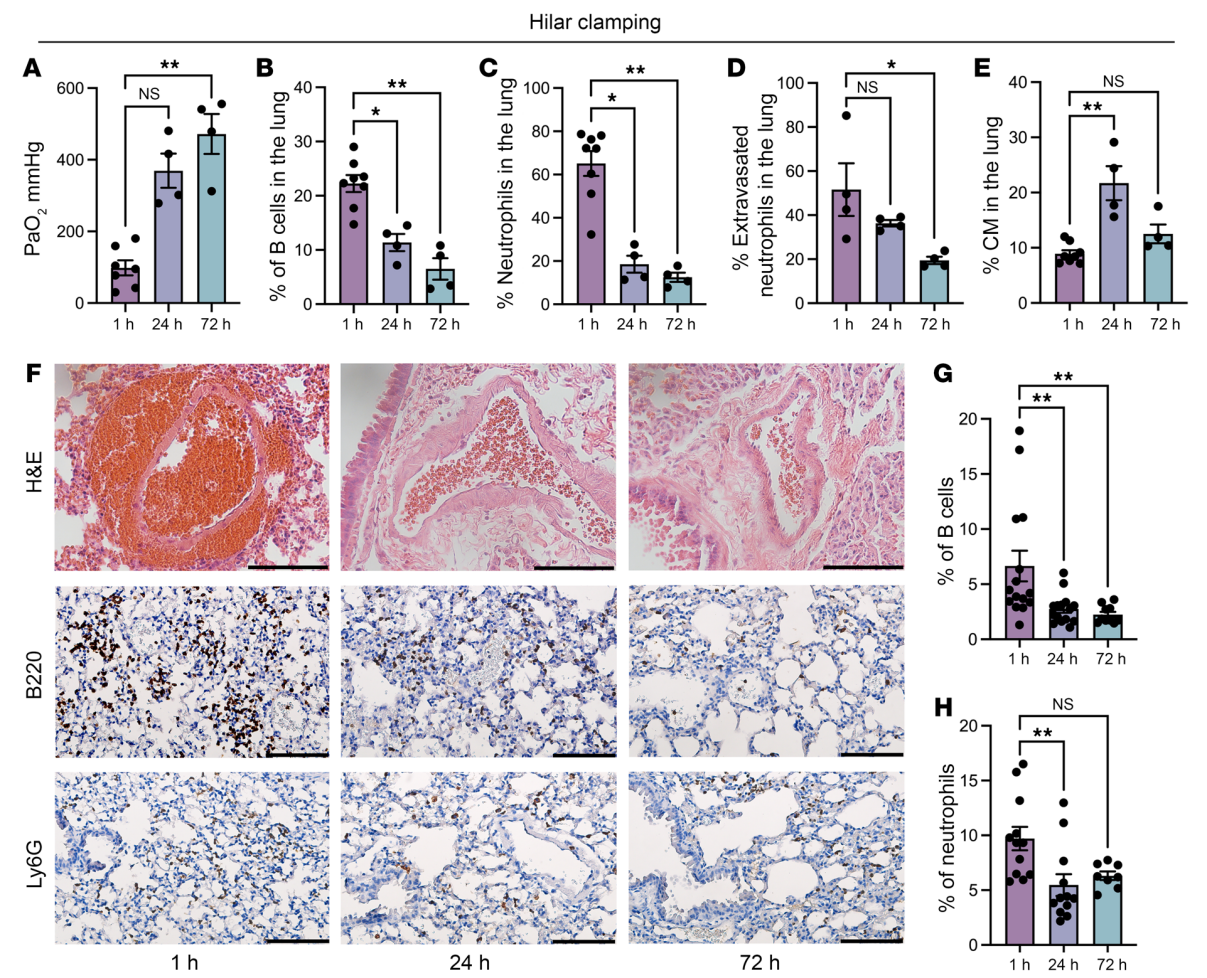
Temporal improvement of lung function after hilar clamping is associated with a gradual and significant reduction in the abundance of B cells in the lung. (**A**) PaO_2_ values after IRI are significantly improved over a period of 72 hours. (**B**–**E**) Flow cytometric analysis of percentages of (**B**) B cells, (**C**) neutrophils, (**D**) neutrophil extravasation, and (**E**) classical monocytes after IRI over time. (**F**) Histological images of hilar clamped lungs from H&E staining and IHC staining for B220 and Ly6G. Immunostaining for B220 represents a separate animal from the same experimental group displayed in Figure 1C. Original magnification, ×40; scale bars**:** 100 μm. (**G** and **H**) Quantification of (**G**) B cells and (**H**) neutrophils per high power field (hpf). Results are presented as mean ± SEM. *n* = 4–8. *P* values were calculated by Kruskal-Wallis test. *P < 0.05, **P < 0.01.

Temporal PaO_2_ assessment after hilar clamping demonstrated that lung injury improves over time (Figure 4A). Flow cytometric analysis showed that this improvement in function is associated with a reduction in the relative abundance of B cells and neutrophils in the lung as well as neutrophil extravasation (Figure 4, B–D). Interestingly, we observed that, in contrast to other cell populations, the proportion of CMs continued to increase (at 24 hours) before decreasing (at 72 hours) to levels comparable to those observed 1 hour after reperfusion (Figure 4E). Histology and IHC staining for B220 and Ly6G also showed that improvement of inflammation correlates with a reduction in the number of B cells and neutrophils in the lung (Figure 4, F–H).

### B cell–derived CCL7 triggers CM recruitment to the lung after IRI.

**Figure 5:**
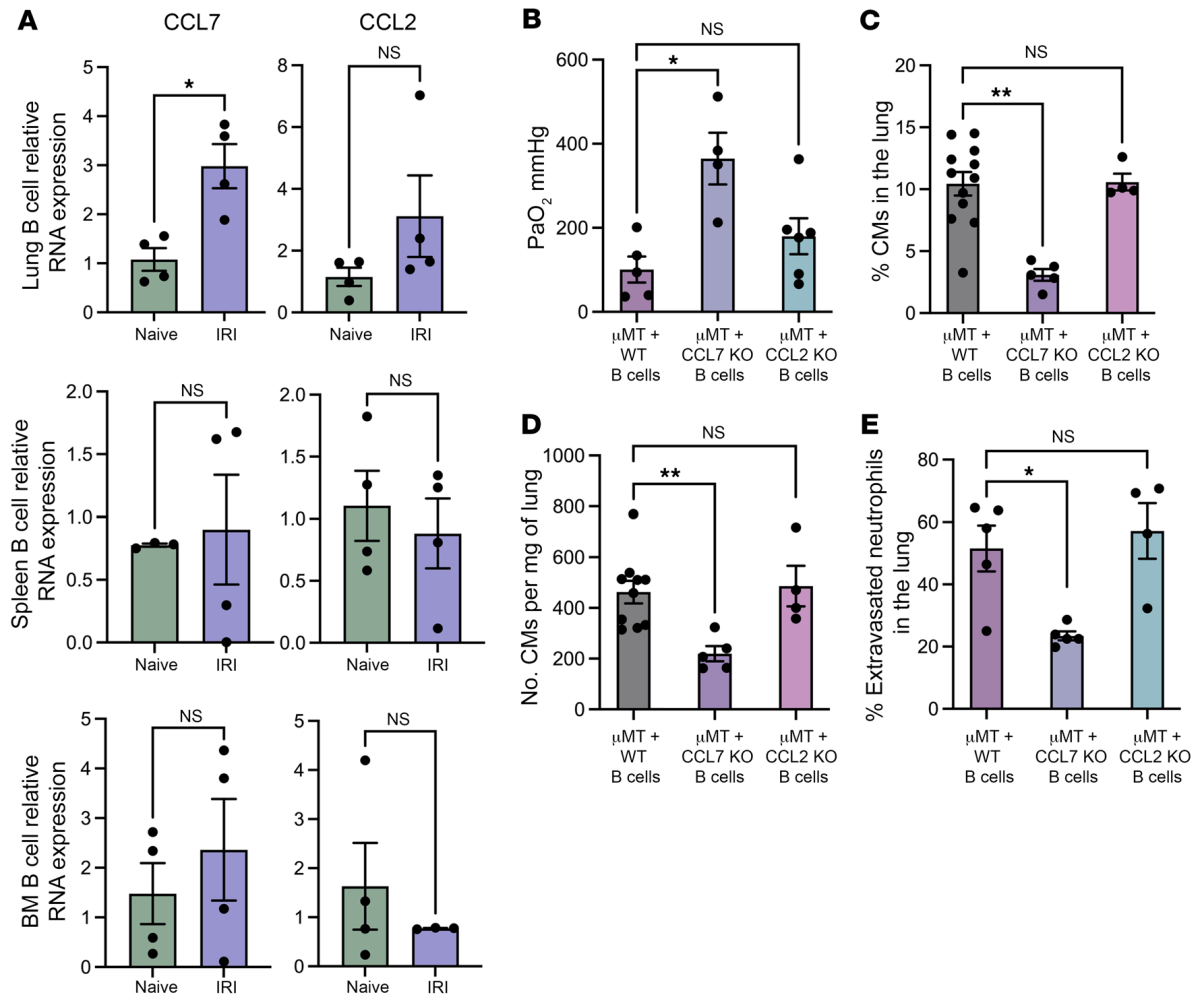
Lung B cell–derived CCL7 production triggers classical monocyte recruitment to the lung after ischemia/reperfusion injury. (**A**) CCL7 and CCL2 relative mRNA expression measured by RT-qPCR in B cells isolated from the lung, spleen, and bone marrow (BM) of B6 WT mice subjected to IRI compared with naive mice. (**B**) Splenic B cells from B6 WT, CCL7^–/–^ or CCL2^–/–^ mice were adoptively transferred to μMT mice 24 hours prior to hilar clamping. Lung function was assessed with ABG. μMT recipients of CCL7^–/–^ B cells showed improved oxygenation. (**C**–**E**) Flow cytometry was used to analyze the (**C**) percentage and (**D**) total number per milligram of lung tissue of classical monocytes and (**E**) percentage of extravasated neutrophils in the lung after IRI. *n* = 4–12. Results are presented as mean ± SEM. (**A**) *P* values were calculated by Mann-Whitney test. *P < 0.05. (**B**–**E**) *P* values were calculated by Kruskal-Wallis test. *P < 0.05, **P < 0.01.

Our group has previously reported that PGD is attenuated after lung transplantation in CCR2-deficient recipient mice, which have defective CM mobilization (25). To gain further insight into how B cells recruit CM to the lung, we examined their production of the monocyte chemoattractant proteins CCL7 and CCL2 after IRI (26, 27). We isolated B cells from the lung, spleen, and bone marrow of naive WT mice and WT mice subjected to left hilar clamping and assessed their expression of CCL7 and CCL2. We observed that lung B cells expressed higher levels of CCL7, but not CCL2, after IRI when compared with B cells in the lungs of naive WT mice. No significant differences were observed in B cell expression levels of CCL2 or CCL7 in either spleen or bone marrow when comparing naive to IRI mice (Figure 5A).

Our group has previously reported that alveolar macrophages facilitate recruitment of CMs to the lung through production of CCL2 (8). Thus, to further investigate the role of CCL7 and CCL2 in CCR2^+^ CM recruitment to the lung after IRI, we treated WT mice with either CCL7- or CCL2-neutralizing antibodies prior to hilar clamping. We observed that oxygenation was significantly improved when CCL7 was inhibited but not when CCL2 was neutralized. We also found a lower percentage and total number of CMs in the lung as well as reduced neutrophil extravasation after CCL7 neutralization when compared with CCL2 and isotype control (Supplemental Figure 2, A–D). We then injected splenic CCL7-deficient (CCL7^–/–^) or WT B cells into μMT mice 24 hours prior to hilar clamping. We observed that oxygenation was significantly improved when μMT mice were reconstituted with CCL7^–/–^ compared with WT B cells, which was associated with significantly lower percentages and total numbers of CMs as well as reduced neutrophil extravasation (Figure 5, B–E). Adoptive transfer of CCL2-deficient (CCL2^–/–^) splenic B cells into μMT mice showed that oxygenation levels, percentages and numbers of CMs and neutrophil extravasation in the lungs were comparable to those in the WT setting (Figure 5, B–E). Taken together, these data show that CCL7 production by B cells mediates CM recruitment to lungs during IRI.

### CM recruitment to the lung after IRI is dependent on TLR4-TRIF signaling in B cells.

**Figure 6:**
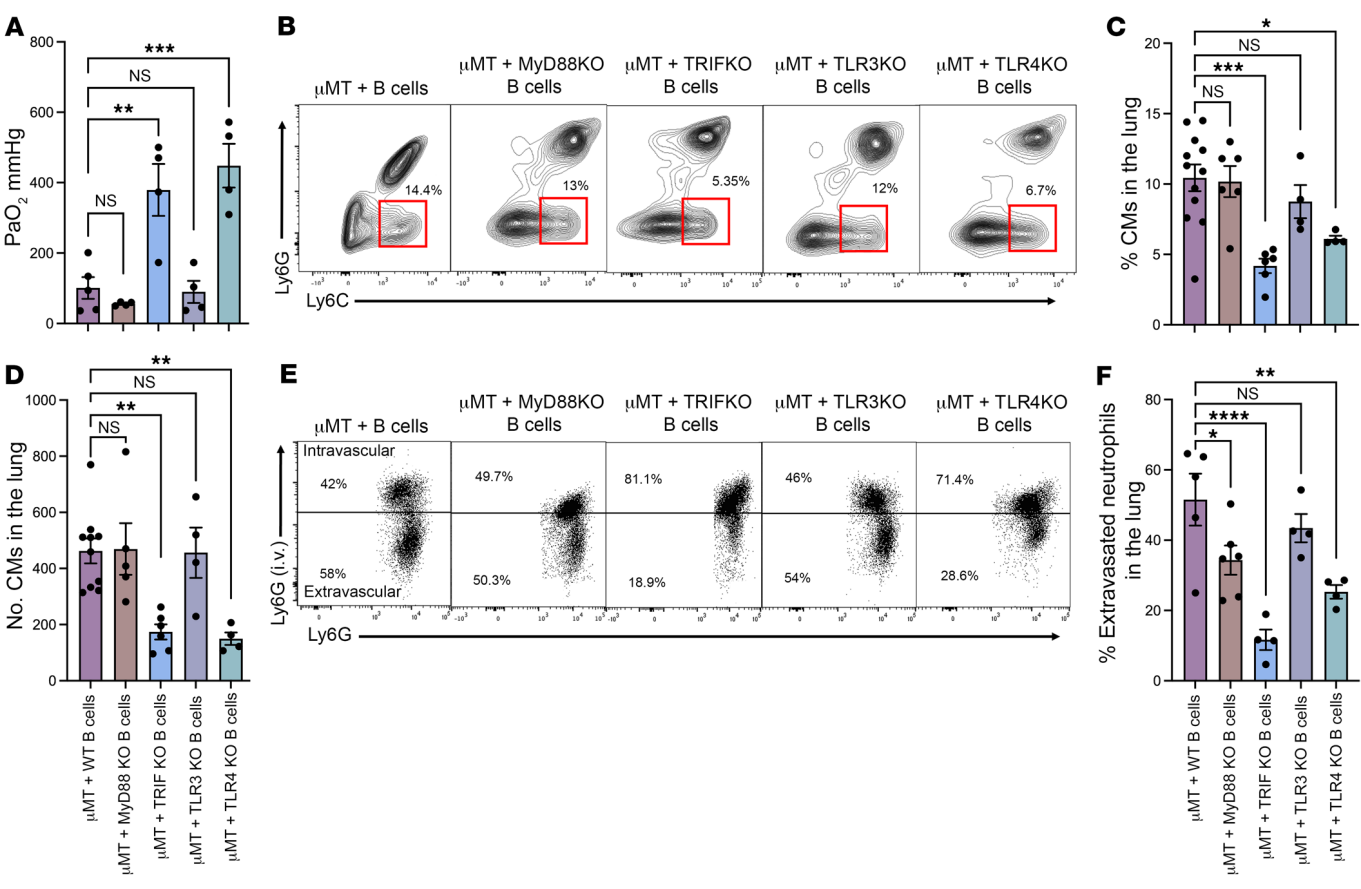
Upstream signaling through TLR4 in lung B cells is required for TRIF-mediated classical monocyte recruitment into the lung after ischemia/reperfusion injury. Splenic B cells from B6 WT, MyD88^–/–^, TRIF^–/–^, TLR3^–/–^, or TLR4^–/–^ mice were adoptively transferred to μMT mice 24 hours prior to hilar clamping. (**A**) Lung function was assessed with ABG. μMT recipients of TRIF^–/–^ and TLR4^–/–^ B cells showed improved oxygenation compared with μMT recipients of B6 WT B cells. (**B**) Flow cytometry contour plots showing the percentage of classical monocytes in the lung. (**C**) Percentage and (**D**) total number per milligram of lung tissue of classical monocytes in above adoptive transfer experiments. (**E**) Flow cytometry dot plots and (**F**) quantification of percentage of extravasated neutrophils in the lung after IRI. *n* = 4–12. Results are presented as mean ± SEM. *P* values were calculated by 1-way ANOVA with post hoc Holm- Šídák test. *P < 0.05, **P < 0.0022, ***P < 0.0002, ***P < 0.0001. μMT + WT B cell data were used as controls for multiple experiments.

B cells express TLRs on their surface, which provides a cell-intrinsic mechanism to respond to innate immune signals (15). We and others have shown that endogenous substances, known as damage-associated molecular patterns (DAMPs), are released from injured cells at the time of transplantation (5, 28, 29). DAMPs can trigger inflammatory responses through innate immune pathways that signal through Toll/IL-IR (TIR) domain-containing adaptor molecules — myeloid differentiation primary response 88 (MyD88) and TIR-domain-containing adaptor-inducing interferon-β (TRIF) (28, 30). To determine whether signaling through MyD88 or TRIF in B cells following IRI has functional consequences, we transferred MyD88-deficient (MyD88^–/–^) or TRIF-deficient (TRIF^–/–^) splenic B cells into μMT mice. We observed that oxygenation was significantly improved in μMT mice that received TRIF^–/–^ cells compared with WT B cells (Figure 6A). Adoptive transfer of MyD88^–/–^ B cells did not result in improvements in lung function. In addition, the percentages and numbers of CMs as well as the proportion of extravasated neutrophils in the lung were decreased after injection of TRIF^–/–^ B cells when compared with WT B cells (Figure 6, B–F).

B cells are known to produce chemokines in response to different TLR signals (16, 31, 32). Within the TLR family, only TLR3 and TLR4 can drive downstream inflammatory responses via TRIF (29). To determine which TLR is responsible for TRIF-mediated B cell activation following hilar clamping, we adoptively transferred splenic B cells from TLR3- (TLR3^–/–^) or TLR4-deficient (TLR4^–/–^) mice into μMT recipients. We observed an improvement in oxygenation in μMT mice that received TLR4^–/–^ but not TLR3^–/–^ B cells compared with WT B cells (Figure 6A). The percentages and numbers of CMs in the lung as well as the percentage of extravasated neutrophils were decreased in mice that received TLR4^–/–^ B cells compared with WT B cells (Figure 6, B–F). Collectively, these data demonstrate that CM recruitment into the lung is dependent on TLR4-TRIF signaling in B cells.

### Lung IRI triggers BCR activation.

**Figure 7:**
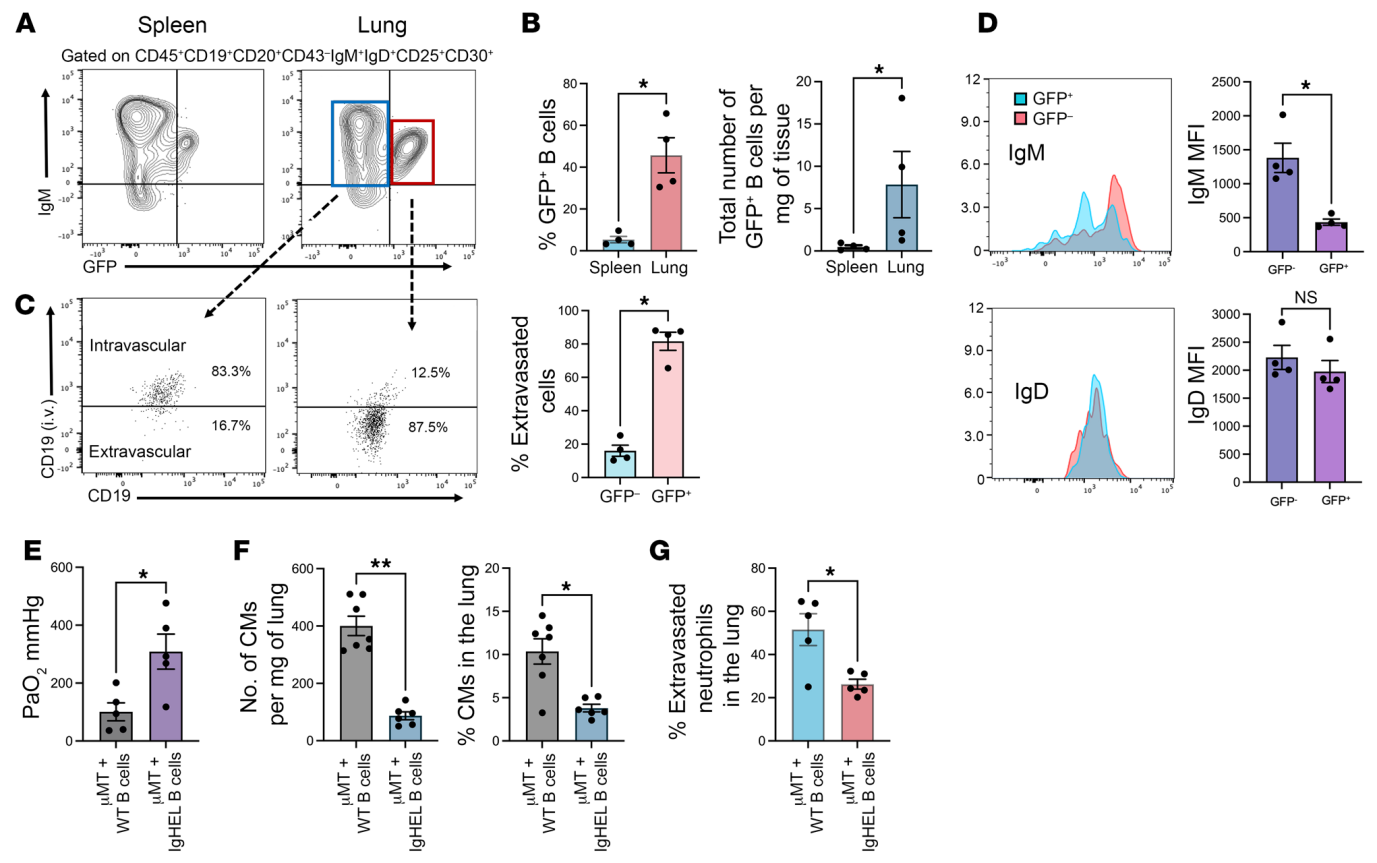
BCR-TLR4 synergistic activation drives classical monocyte recruitment to the lung after IRI. In Nur77^GFP^ mice, there is GFP expression when the BCR is activated. (**A**) Flow cytometry gating strategy for GFP expression on activated B cells (CD25^+^ CD30^+^) in spleen and lung after hilar clamping of Nur77^GFP^ mice. (**B**) Percentage and total number of GFP^+^ B cells per milligram of lung tissue. (**C**) Representative dot plots of flow cytometric analysis of B cell extravasation, which was determined by injecting fluorochrome-labeled B cell–specific anti-CD19 antibodies i.v. 5 minutes before sacrifice. The graph shows the mean percentage of B cell extravasation in the GFP^–^ versus GFP^+^ populations. (**D**) IgM and IgD mean fluorescence intensity measured on lung-infiltrating GFP^+^ and GFP^–^ B cells. (**E**) Splenic B cells from IgHEL mice were adoptively transferred to μMT mice 24 hours prior to hilar clamping. Lung function was assessed with ABG. μMT recipients of IgHEL B cells showed improved oxygenation compared with B6 WT B cells. (**F** and **G**) Flow cytometry was used to analyze the (**F**) percentage and total number per milligram of lung tissue of classical monocytes and (**G**) percentage of extravasated neutrophils in the lung after IRI. *n* = 4–7. Results are presented as mean ± SEM. *P* values were calculated by Mann-Whitney test. *P < 0.05, **P < 0.01.

B cells express both TLRs and BCRs on their surface, and the downstream signaling of these receptors coordinates the B cell response to pathogen challenge as well as B cell survival, development, and antibody production under both physiological and pathological conditions (33). B cells express CD25 and CD30 on their surface after activation, and the expression of CD25 increases following TLR-mediated stimulation (34–36). To determine whether the BCR is activated after lung IRI, we hilar-clamped Nur77^GFP^ mice (B cells express GFP when their BCR recognizes antigen) (37) and measured GFP expression in activated B cells (CD25^+^CD30^+^). We observed an increased proportion and total number of CD25^+^CD30^+^ B cells expressing GFP in the lung compared with the spleen, indicating that there is endogenous antigen recognition by the BCR in these activated B cells in the injured lungs (Figure 7, A and B). Interestingly, a higher percentage of GFP^+^ B cells was extravasated compared with GFP^–^ B cells (Figure 7C). It has been shown that both immune (antigen-induced) and nonimmune (natural) IgM play a role in responses against pathogens and self-antigens (31). Naive B cells coexpress 2 BCR isotypes, IgM (monomer) and IgD (pentamer), with identical antigen-binding domains but distinct constant regions. These two receptors perform similar roles and can largely substitute for one another. The prevailing view is that IgM is more sensitive in recognizing “self” compared with IgD; B cells that identify self-antigens therefore decrease their surface IgM levels (38). We measured the mean fluorescence intensity (MFI) of IgM and IgD on lung GFP^+^ and GFP^–^ B cells. Indeed, we found that lung GFP^+^ B cells had a lower IgM MFI than lung GFP^–^ B cells but maintained similar IgD MFI levels (Figure 7D).

We hypothesized that synergistic BCR and TLR4 activation on B cells is required to mediate lung IRI and investigated whether blocking BCR activation has functional consequences. We adoptively transferred splenic B cells from IgHEL mice (39) (where the BCR is not activated unless it is stimulated by a specific antigen, hen egg lysozyme) into μMT mice 24 hours prior to hilar clamping. We observed improved oxygenation, decreased abundance of CMs, and reduced neutrophil extravasation in the lungs of μMT mice that received IgHEL compared with WT B cells (Figure 7, E–G). Taken together, these data suggest that the BCR on lung-infiltrating B cells recognizes endogenous antigens released during IRI and that the ability of B cells to mediate CM recruitment depends on their concomitant activation of TLR4 and their BCR.

### Recipient-derived B cells mediate CM recruitment and worsen IRI after murine lung transplantation.

**Figure 8:**
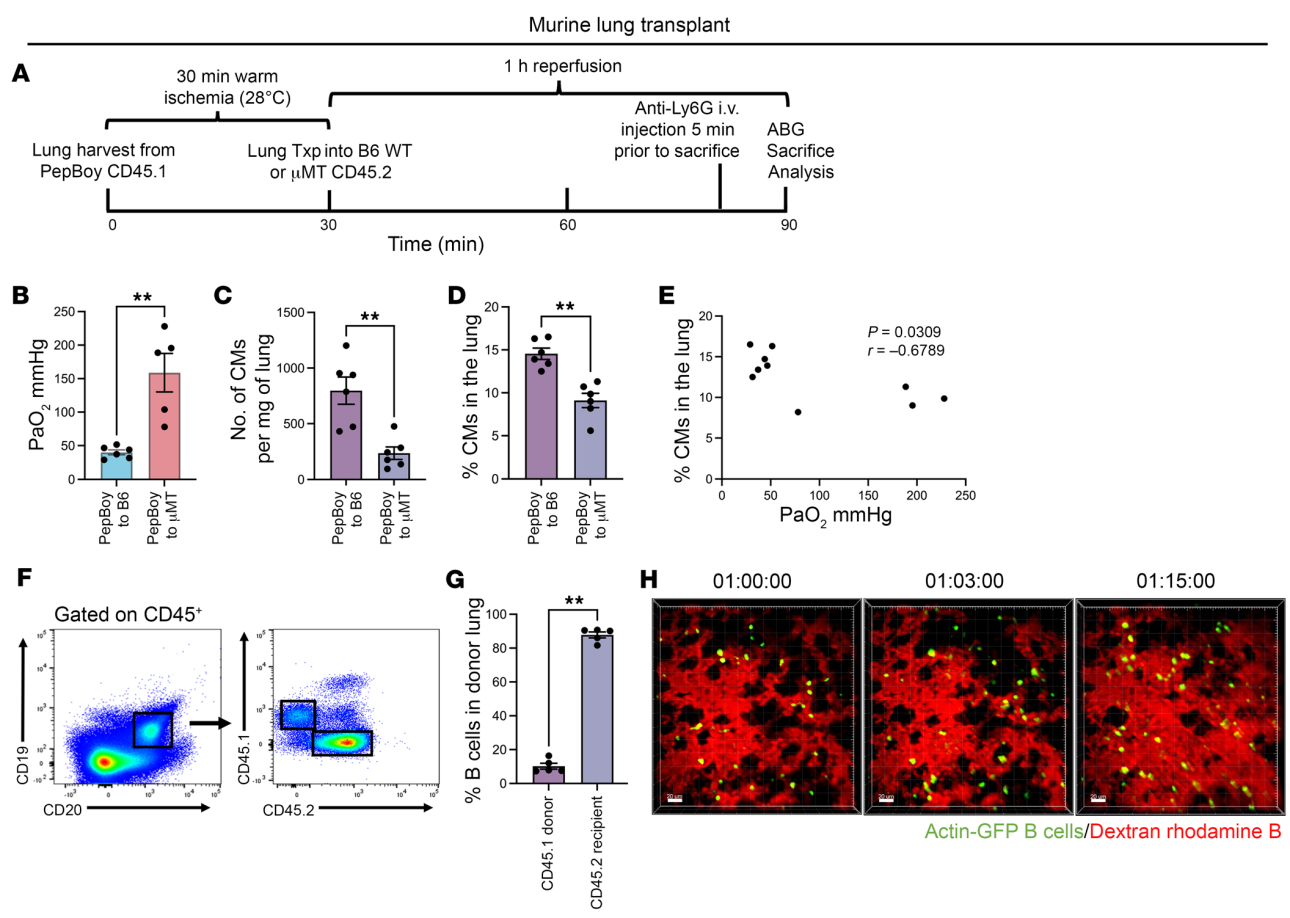
Recipient-derived B cells increase classical monocyte recruitment, thereby worsening lung function after syngeneic murine lung transplantation. (**A**) To distinguish between tissue-resident donor and graft-infiltrating recipient-derived B cells, we transplanted B6 CD45.1 donor lungs subjected to 30 minutes of warm ischemia (28°C) into B6 WT or μMT recipient (CD45.2) mice. Donor lung graft was harvested 1 hour after reperfusion, and flow cytometry was used to analyze cell populations. Txp, transplant. (**B**) PaO_2_ values showed improved oxygenation in μMT recipients compared with B6 WT CD45.2 recipients. (**C**) Total number per milligram of lung tissue and (**D**) percentage of classical monocytes are higher in the μMT recipients compared with B6 WT recipients. (**E**) Negative correlation between percentage of classical monocytes and PaO_2_ values after IRI. Pearson’s correlation coefficient (*r*) was significant. (**F**) Representative flow cytometry analysis of lung graft differentiating donor- vs. recipient-derived B cells. (**G**) Quantification of CD45.1 (donor) vs. CD45.2 (recipient) B cells after syngeneic lung transplant. Results are presented as mean ± SEM. *n* = 4–6. *P* values were calculated by Mann-Whitney test. **P < 0.01. (**H**) Time-lapse intravital 2-photon imaging at 1 hour after engraftment showing graft-infiltrating B cells entering the pulmonary vasculature over time. Dextran-rhodamine B was used to label blood vessels. Scale bar: 20 μm. Images are representative of 2 independent experiments with comparable results.

In order to study the role of B cells in a more clinically relevant model of IRI, we performed syngeneic murine left lung transplants (40). We transplanted B6.SJL-*Ptprc^a^ Pepc^b^*/BoyJ (PepBoy) lungs (CD45.1) into congenic B6 WT or μMT mice (CD45.2), which allowed us to differentiate the contributions of donor- (CD45.1) versus recipient- (CD45.2) derived B cells to IRI. Donor lungs were harvested and exposed to warm ischemia (28°C) for 30 minutes prior to transplantation, as previously described (40). Lungs were analyzed 1 hour after engraftment (Figure 8A). We observed that oxygenation was significantly improved and the number and percentage of CMs were decreased after transplantation into μMT compared with WT recipients (Figure 8, B–D). Greater CM recruitment to the lung graft was associated with lower oxygenation levels (Figure 8E). Additionally, we found that the vast majority (~90%) of B cells that are present in lung grafts 1 hour after transplantation were of recipient origin (Figure 8, F and G). Our group has previously used 2-photon microscopy to study leukocyte dynamics in vivo (5, 6, 41). We performed intravital microscopy to further examine B cell recruitment into a transplanted lung. We transferred splenic B cells from β-actin–GFP mice to syngeneic lung transplant recipients (B6 WT to B6 WT). Two-photon microscopy revealed many B cells entering the pulmonary vasculature from the periphery immediately after engraftment. Some of the B cells could be seen flowing rapidly through the vessels, while other cells transited more slowly (Figure 8H and Supplemental Video 1). Consistent with our flow cytometric results, we also observed that some B cells had extravasated into the lung parenchyma (Supplemental Video 2). These findings indicate that recipient-derived B cells play an important role in mediating CM recruitment and tissue damage following lung transplantation.

We also performed temporal analysis of lung grafts after syngeneic lung transplantation. We found that similar to hilar clamping, lung function and inflammation improve over time (Supplemental Figure 3). Even though we did not find a difference in the number of B cells, we observed that the number of neutrophils (Ly6G) within graft significantly decreased over time (Supplemental Figure 3, B–D). For both syngeneic and allogeneic transplants, we found that graft function was comparable between B6 and μMT recipients at 24 hours after reperfusion (Supplemental Figure 4, A and B). We also observed that the percentage of CMs in grafts and neutrophil extravasation were decreased in μMT in both conditions. Lung-restrictive antibodies (LRAs) have been associated with a higher incidence of PGD after murine syngeneic lung transplantation (42, 43). Thus, we investigated whether LRAs are secreted by B2 B cells after hilar clamping. Interestingly, we found that LRA production against collagen V was elevated in both B6 WT and μMT mice compared with naive mice. This suggests that other B cell populations present in μMT mice (e.g., B1 B cells) could be responsible for the production of LRAs (Supplemental Figure 5A). Interestingly, complement 4d (C4d) deposition was seen in lungs only after allogeneic transplantation but not after hilar clamping or syngeneic transplantation (Supplemental Figure 5, B and C).

### Transcriptional diversity exists in recipient lung B cells following syngeneic transplantation.

**Figure 9:**
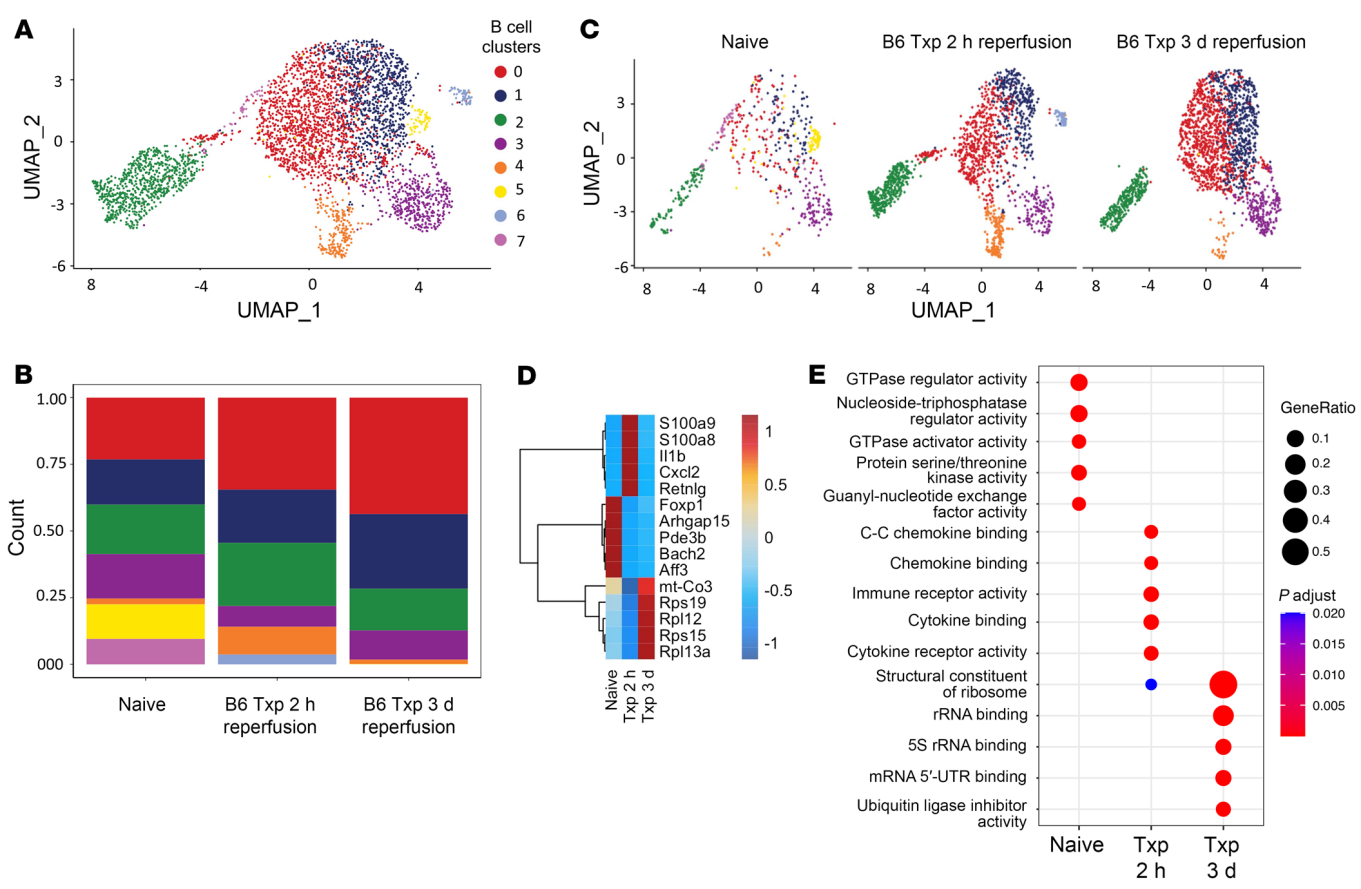
Single-cell RNA-Seq characterization of recipient B cell infiltration following syngeneic mouse lung transplantation. Syngeneic left lung transplants with 60 minutes of cold ischemia and 45 minutes of warm ischemia followed by either 2 or 72 hours of reperfusion were performed. Recipient cells were sorted from the left lung by fluorescence-activated cell sorting (FACS) and processed for single-cell RNA-Seq (scRNA-Seq). hr, hour. (**A**) UMAP embedding plot of recipient B cell states. (**B**) UMAP embedding plot of B cell states split by time after transplant. (**C**) Recipient B cell state composition stack plot grouped by time after transplant. (**D**) Heatmap of most highly differentially expressed genes in recipient B cells across time points after transplant; scaled by row. (**E**) Gene Ontology pathway analysis using statistically significant differentially expressed genes across time points after transplant (adjusted *P* < 0.05 and average log_2_FC > 0.58). *P* values were calculated by Wilcoxon’s rank-sum test.

To characterize the transcriptional landscape of B cells following warm ischemia in syngeneic lung transplantation, we performed single-cell RNA-Seq (scRNA-Seq) in naive and transplanted lungs 2 hours and 3 days after transplantation. We sorted recipient (CD45.1) cells for scRNA-Seq and, after quality control, dimensional reduction, and cluster annotation, we focused on B cell states (Figure 9A). Specifically, we identified 8 transcriptionally distinct B cell states (Figure 9B). Cell composition analysis showed that the postischemic lung had unique recipient B cell states compared with naive lungs. In particular, cluster 0 expanded over time and cluster 4 was most enriched at 2 hours compared with naive lungs and grafts 3 days after transplantation (Figure 9, B and C). In naive lungs, B cells highly expressed transcription factors involved in B cell differentiation, such as *Foxp1* and *Bach2* (Figure 9D). At 2 hours after reperfusion, recipient B cells highly expressed alarmins (*S100a8*, *S100a9*), proinflammatory cytokines (*Il1b*), and interestingly, a neutrophil chemoattractant *Cxcl2* (Figure 9D). At 3 days after reperfusion, the most strongly expressed genes were ribosomal, suggesting active protein production (Figure 9D). Correspondingly, pathway analysis revealed that immune receptor binding and activation was pronounced at 2 hours and ribosomal RNA binding was highest at 3 days after reperfusion (Figure 9E). Collectively, our results show that ischemia in the setting of lung transplantation leads to a temporal diversification of recipient B cells with functionally distinct cell states in early and late phases of injury.

### The presence of B cells correlates with higher levels of CM recruitment and neutrophilic extravasation after human lung transplantation.

**Figure 10:**
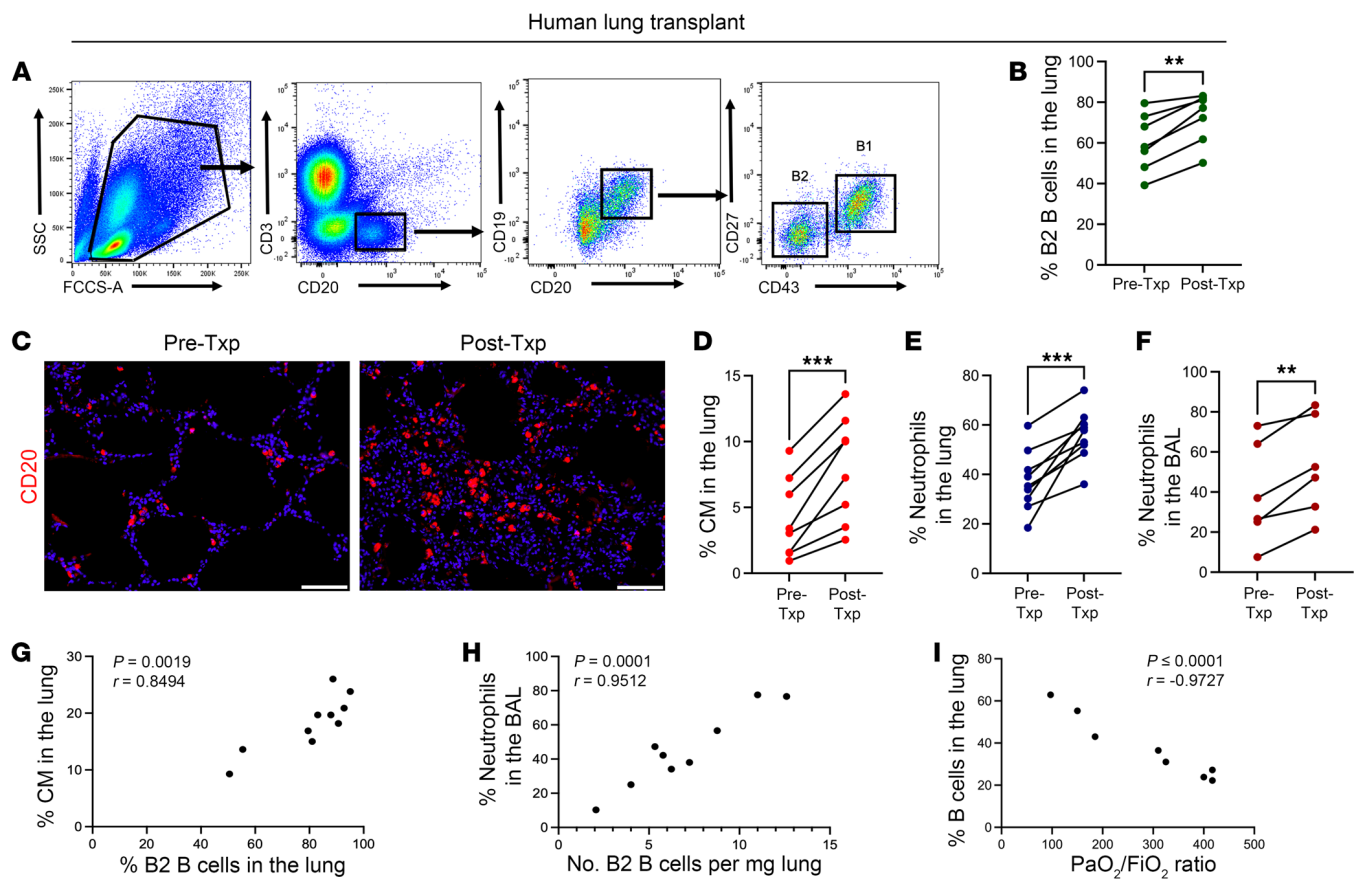
Higher numbers of B cells correlate positively with the number of classical monocytes and neutrophil extravasation in the lung following human lung transplantation. Specimens were collected from human donor lungs at the time of back-table preparation (Pre-Txp) and at 2 hours after reperfusion (Post-Txp). Flow cytometry was used to analyze cell populations. (**A**) Flow cytometry gating strategy for human B2 B cells (CD20^+^CD19^+^CD43^–^CD27^–^) and B1 B cells (CD20^+^CD19^+^CD43^+^CD27^+^). (**B**) Proportion of B2 B cells in the lung before and 2 hours after reperfusion. (**C**) Immunofluorescence staining of human lung tissue for CD20 expression before and after lung transplantation. Original magnification, ×20; scale bars: 200 μm. Images are representative of 2 independent experiments with comparable results. (**D**) Percentage of classical monocytes, (**E**) neutrophils in the lung, and (**F**) neutrophils in BAL (extravasated neutrophils) before and 2 hours after reperfusion. (**G**) Positive correlation between percentage of classical monocytes and percentage of B2 B cells in the lung. (**H**) Positive correlation between percentage of neutrophils in the BAL and the number of B2 B cells in the lung. (**I**) Inverse correlation between the percentage of B cells in the lung after reperfusion and the PaO_2_/FiO_2_ ratio of the transplant recipients up to 72 hours after transplantation. (**G**–**I**) Pearson’s correlation coefficients (*r*) were significant. *n* = 6–10. (**B** and **D**–**F**) *P* values were calculated by paired *t* test. **P < 0.01, ***P < 0.001.

To correlate our findings in mice to human lung transplants, we collected lung specimens at the time of back-table preparation (before transplant) and approximately 2 hours after reperfusion (after transplant). In humans, B2 B cells are defined as CD20^+^CD19^+^CD43^–^CD27^–^, B1 B cells as CD20^+^CD19^+^CD43^+^CD27^+^ (Figure 10A), neutrophils as CD45^+^CD11b^+^CD15^+^, and CMs as CD45^+^CCR2^+^CD11b^+^CD14^++^CD16^–^ (Supplemental Figure 6, A–C). We observed a significantly increased abundance of B2 B cells on both flow cytometry and immunostaining after transplant compared with before transplant (Figure 10, B and C). We also observed an increase in abundance of CMs and neutrophils in the lung parenchyma (Figure 10, D and E). The presence of neutrophils in the bronchoalveolar lavage (BAL) fluid is a surrogate for neutrophil extravasation. Examination of recipient BAL fluid demonstrated a larger proportion of neutrophils 2 hours after transplant compared with that before transplant (Figure 10F and Supplemental Figure 6B). We found a positive correlation between the percentages of CMs and B cells in the lung graft as well as a positive correlation between the number of B cells in lung graft and the percentage of neutrophils in the BAL (Figure 10, G and H). Most importantly, we identified an inverse correlation between the number of B cells in the lung after reperfusion and the PaO_2_/FiO_2_ ratio of transplant recipients up to 72 hours, suggesting that increased abundance of B cells is associated with worsened lung allograft function (Figure 10I). These data extend our findings in mice and suggest that B cells could play a similar role in mediating CM recruitment and neutrophil extravasation following human lung transplantation.

## Discussion

Using murine lung hilar clamping and transplant models of IRI, we show that B cells are critical early contributors to the recruitment of CCR2^+^ CMs into the lung, which then cause neutrophil extravasation and subsequent organ dysfunction. Importantly, we corroborate these findings in human lung transplant recipients. Most notably, we found that the abundance of B cells correlates inversely with postoperative lung function in recipients, expressed as lower postoperative PaO_2_/FiO_2_ ratio.

Mechanistically, we demonstrate that recipient-derived B cells traffic to the lung very shortly after reperfusion. The ensuing inflammatory response in the graft then results in rapid activation of both TLR4-TRIF and BCRs on B cells, which drives their production of the monocyte chemokine CCL7. Previous studies of heart and kidney IRI have shown that B cell–derived CCL7 increases monocyte recruitment (20, 21), a finding which we corroborate in the lung. However, the cell-intrinsic mechanism responsible for B cell activation and CCL7 production after lung IRI has not been previously described to our knowledge. We propose that the release of specific local signals (e.g., DAMPs) (44) after ischemic injury results in rapid TLR4 engagement on lung-infiltrating B cells. Signaling through TRIF leads to the production of inflammatory cytokines such as type I interferons and results in the upregulation of B cell activation markers, proliferation, cytokine and immunoglobulin secretion, and terminal differentiation (45–47). Our adoptive transfer experiments demonstrated that TLR4 signaling through TRIF in B cells mediates CM recruitment to the injured lung after IRI.

Neutrophils critically mediate the extensive inflammatory response following IRI, but their trafficking mechanisms vary by organ (5, 7, 41, 48, 49). Upon arrival at the site of injury, neutrophils exacerbate lung damage via production of reactive oxygen species, formation of neutrophil extracellular traps, and blockade of capillaries preventing tissue reperfusion, leading to loss of endothelial barrier integrity and release of proinflammatory cytokines (29, 50–52). Furthermore, warm ischemia exacerbates IRI in both clinical and experimental settings (53). While depletion of neutrophils attenuates IRI in several animal models, the critical role that they play in host defense makes global neutrophil depletion strategies to reduce IRI not clinically practical (54, 55). Neutrophils were thought to be the first innate immune cell type to enter the lung during infection or sterile inflammation; however, studies from our group have demonstrated that monocyte extravasation temporally precedes neutrophil extravasation in the setting of transplant-induced inflammation (6, 7). Our current results extend these observations by demonstrating that B cells act even further upstream of monocytes during lung IRI. This finding suggests that B cells may be targeted to decrease lung dysfunction following IRI.

In a mouse lung transplant model with warm IRI, recipient B cells have been shown to contribute to chronic lung allograft rejection, airway and parenchymal fibrosis, and formation of circulating autoantibodies (56). We build upon these findings by demonstrating on intravital 2-photon microscopy that rapid recruitment of recipient B cells occurs within 1 hour of reperfusion. It is worth noting that, while the majority of B cells in the allograft are recipient-derived, there is also a small population of donor B cells. Donor-derived B cells have been shown to play a protective role against IRI, possibly via downregulation of donor neutrophil and monocyte numbers (57). While our current study conclusively shows that recipient B cells potentiate IRI, the role of donor B cells needs to be explored in future studies.

Clinically, PGD occurs within the first 72 hours after transplantation. PaO_2_ readings attributed to the left lung improve by 24 hours after reperfusion in both the hilar clamp and transplant models, suggesting that some degree of recovery occurs after the immediate insult of IRI. Both B cell and neutrophil abundance is highest at 1 hour after reperfusion and declines over time. This could in part be explained by the immediate increase in vascular permeability seen with acute lung inflammation, which improves over time. Interestingly, we see a higher number of CMs in the lung at 24 hours after reperfusion as compared with 1 hour and 72 hours (Figure 4E and Supplemental Figure 7). We speculate that the B cell–CM-neutrophil axis potentiates IRI in the early hours following reperfusion, but the driver of monocyte recruitment may shift to another cell type by 24 hours. CCR2 on CMs binds two monocyte chemoattractants, CCL2 and CCL7, which facilitate the release of CMs from the bone marrow into the circulation and to sites of inflammation (58, 59). Our group has previously published that, at 24 hours after transplant, alveolar macrophages facilitate CM recruitment to the injured lung through their production of CCL2 (8). In this study, B cell–derived CCL7, but not CCL2, is necessary for CM recruitment at 1 hour after reperfusion. Thus, it appears that CCL7 produced by B cells plays an earlier role in the recruitment of CMs, while CCL2 production by alveolar macrophages may become more critical at later time points.

Allogeneic transplantation showed that recipient B cells were similarly associated with CM recruitment and neutrophil extravasation as in syngeneic transplants. The main difference was the presence of C4d deposition in allogeneic transplants, which was absent in the setting of hilar clamping and syngeneic transplantation. Thus, it seems that recipient B cells recruit CMs to the lung graft irrespective of the presence of alloantigens. However, the presence of alloantigens may predispose to C4d deposition in grafts (8). While we showed that these early graft-infiltrating B cells are not instrumental in secreting LRAs (43), whether they produce alloantibodies responsible for complement deposition and antibody-mediated rejection is unclear and warrants further investigation.

We have established an innate immune role played by B cells in the recruitment of CMs after IRI, which depends on concomitant activation of the BCR and TLR4 receptors shortly after reperfusion. We speculate that the dual engagement of BCR and TLR4 on B cells at 1 hour may link their innate and later adaptive immune functions (60). Once antigen bound, BCRs are internalized, and the activated B cell can present antigen to T cells (61) — this corresponds to the decreased surface IgM levels we observed on lung B cells after IRI. Additionally, BCR activation alone cannot induce CSR without costimulation provided either by TLR4 ligands or by CD40L (62–64). Thus, it is possible that the early coactivation of BCR and TLR4 on B cells following IRI facilitates antigen-specific CSR to produce high-affinity antibodies and generation of immunological memory.

We postulate that DAMPs/endogenous antigens released from tissue injury following a period of ischemia could bind both TLRs and BCRs on B cells after reperfusion (44). Possible candidate TLR4 ligands include HMGB1 (65–67), heat shock proteins, hyaluronan, fibrinogen, and S100 proteins (68). Identification of the specific DAMP(s) responsible for B cell activation is an important area for future investigation. Another question that warrants further exploration is the location of B cell antigen binding. We observed that most of the activated B cells are extravascular, which raises the question of whether intravascular antigen exposure facilitates B cell extravasation or B cell extravasation is a prerequisite for intraparenchymal antigen exposure. It has been demonstrated that intravascular NCM antigen presentation to CD4^+^ T cells is required for subsequent T cell migration across endothelial layers (69, 70).

In our murine hilar clamp model, we have demonstrated the efficacy of preischemia depletion of B cells with anti-CD20 neutralizing antibody in reducing subsequent CM recruitment and improving lung oxygenation. Human monoclonal anti-CD20 therapies (i.e., rituximab) exist and are used to treat B cell lymphomas and posttransplant lymphoproliferative disorders (71). Ku et al. have demonstrated effective B cell depletion by delivering rituximab to human donor lungs using ex vivo lung perfusion (72). A randomized controlled trial demonstrated that rituximab induction therapy may reduce early donor-specific antibody production in pediatric lung transplant recipients (73). Even more recently, chimeric antigen receptor T cell (CAR T cell) therapies have been used to target B and plasma cells in the treatment of large B cell lymphoma and multiple myeloma (74, 75). In fact, there is an upcoming clinical trial investigating the use of CAR T cell depletion of B and plasma cells in kidney transplant candidates with high panel-reactive antibodies (i.e., preexisting alloantibodies), with the goal of desensitizing these patients and improving donor-recipient matching (76). We acknowledge that global depletion of B cells could also affect regulatory phenotypes, and depletion of the latter may have deleterious effects on graft tolerance (77). Interestingly, we found at least 8 genetically distinct subsets of B cells that infiltrate the lung after reperfusion based on scRNA-Seq data. There is immense therapeutic potential in gaining a better understanding of the identity and fate of these B cell subsets, which will be the focus of future investigation.

In summary, our study uncovers a role for B cells in the pathogenesis of lung IRI. We introduce a mechanism by which lung-infiltrating B cells initiate a cell-intrinsic signaling cascade that results in CM recruitment and neutrophil-mediated lung dysfunction following IRI. Additionally, we show that this signaling cascade is triggered by the dual activation of both the BCR and TLR4 receptors on B cells, which may have implications for the development of later adaptive immune responses. Our study adds to the existing understanding of immune mechanisms that mediate transplant-associated IRI (5, 7, 8, 41, 78–80) and lays the foundation for development of B cell–targeting therapies in the perioperative period of lung transplantation (81, 82).

## Methods

### Sex as a biological variable

Our study examined male and female animals, and similar findings are reported for both sexes.

### Human samples

Donor lung specimens were obtained at 2 time points: (a) during back-table preparation of the donor block and (b) approximately 120 minutes after graft reperfusion. Lung specimens were transported to the laboratory in cold saline and processed for flow cytometry. BAL fluid samples were obtained via bronchoscopy from the recipient before the start of the lung transplant procedure and 2 hours after implantation/reperfusion. ABGs were collected from transplant recipients within 72 hours after transplantation.

### Mice and procedures

#### Mice.

WT C57BL/6J (B6), B6.129S2-Ighm*^tm1Cgn^*/J (μMT), BALB/cJ (BALB/c), B6.129S4-Ccl2*^tm1Rol^*/J (CCL2^–/–^), B6.129S4-Ccl7*^t1lfcl^*/J (CCL7^–/–^), B6.129S1-Tlr3*^tm1Flv^*/J (TLR3^–/–^), B6(74)-Tlr4^tm1.2Karp^/J (TLR4^–/–^), C57BL/6J-Ticam1*^Lps2^*/J (TRIF^–/–^), B6.129P2(SJL)-Myd88*^tm1.1Defr^*/J (MyD88^–/–^), B6.SJL-*Ptprc^a^ Pepc^b^*/BoyJ (Pepboy or B6 CD45.1), C57BL/6-Tg(Nr4a1-EGFP/cre)820Khog/J (Nur77^GFP^), C57BL/6-Tg (IghelMD4)4Ccg/J (IgHEL), and C57BL/6-Tg(CAG-EGFP)131Osb/LeySopJ (β-actin-GFP) mice were obtained from The Jackson Laboratory. All mice were maintained in a dedicated pathogen-free animal facility at Washington University and were used for the described experiments at 8–10 weeks of age. Male and female mice were used randomly.

#### Lung hilar clamping.

Mice were anesthetized with intraperitoneal injection of ketamine (80–100 mg/kg of body weight) and xylazine (8–10 mg/kg of body weight), intubated orotracheally with a 20 G angiocatheter connected to a small-animal ventilator (Harvard Apparatus). The animals were secured on a thermal pad at 28°C and maintained under general anesthesia with 1.2%–1.7% isoflurane at 1 mL/min and with fraction of inspired oxygen of 100%. A left thoracotomy in the fourth intercostal space was performed and a rib retractor was placed. The inferior pulmonary ligament was divided, and the hilum was dissected. The pulmonary artery, pulmonary veins, and main stem bronchus were occluded securely with releasable 4-0 silk suture ligation for 30 minutes. Following 30 minutes of ischemia, the hilar ligation suture was released, allowing the left lung to reperfuse for 60 minutes. Fluorochrome-labeled antibody (anti-Ly6G, anti-Ly6C, or anti-CD19) was injected into the IVC 5 minutes prior to sacrifice. This allowed for subsequent measurement of neutrophil, CM, and B cell extravasation, respectively, into the lung parenchyma. For selected experiments, mice were treated with 25 ng anti-CCL7 antibody (AF-456-NA, R&D Systems) or 25 ng anti-CCL2 antibody (AF-479-NA, R&D Systems) i.v. 24 hours prior to hilar clamp. For B cell depletion, anti-mouse CD20 antibody (clone MB20-11, Bio X cell) (200 μg i.v. + 200 μg i.p.) was administered 4 days prior to hilar clamping.

#### ABG assessment.

The laparotomy was extended into bilateral clamshell thoracotomy. The right pulmonary hilum was dissected and occluded with 4-0 silk suture ligation at 4 minutes prior to the end of reperfusion. At the end of the 60-minute reperfusion period, approximately 200 μL arterial blood was aspirated from the left ventricle. ABG was measured using an iSTAT Portable Clinical Analyzer (iMALE STAT Corp). The left lung was then harvested for analysis.

#### Murine lung transplantation.

Orthotopic left lung transplantation was performed from a Pepboy or BALB/c donor into a congenic B6 WT or μMT mouse, as described previously (40). Following donor lung harvest, the graft was kept in a water bath at 28°C for 30 minutes of warm ischemia. Follow graft implantation into the recipient, the lung was reperfused for 1 hour prior to sacrifice. ABG was obtained prior to the end of reperfusion as described above.

#### Two-photon intravital microscopy.

B cells (~3 × 10^6^) isolated from β-actin–GFP mice were injected i.v. into the recipient of a syngeneic left lung transplant with 30 minutes of warm ischemia followed by 1 hour of reperfusion, as described above. One hour after reperfusion, mice were anesthetized and reintubated. 8 μL dextran-rhodamine B (30,000MV, Invitrogen), suspended in 150 μL phosphate-buffered saline was injected i.v. to label blood vessels. Mice were placed in right lateral decubitus position; the thoracotomy was reopened and left third to sixth ribs were resected to create a small left thoracic window. Lungs were imaged with a custom-built 2-photon microscope using SlideBook acquisition software (A&B Software). For time-lapse imaging, we averaged 22 video-rate frames (0.75 seconds per slice) during the acquisition. Each plane represents an image of 220 × 240 μm in the *x* and *y* dimensions (6).

### Multicolor flow cytometry

Lung tissue was digested in RPMI 1640 solution containing type 1 collagenase (0.5 mg/mL) (Worthington Biochemical Corporation) and 5 U/mL DNase I (MilliporeSigma) for 60 minutes. Single suspension was then created by passing the digested lung and spleen tissue through a 70-μm strainer, followed by erythrocyte lysis with Ammonium-Chloride-Potassium lysing buffer (Quality Biological), and then passing final cells through a 40-μm strainer. Cells were Fc blocked for 15 minutes using anti-mouse CD16/CD32 (clone 93, Invitrogen) prior to surface antibody staining. Flow antibodies against mouse included CD45 (clone 30-F11, Thermo Fisher Scientific; clone 30-F11, BD Biosciences), CD19 (clone eBio1D3[1D3], Thermo Fisher Scientific; 1D3, BD Biosciences), CD45R (clone RA3-6B2, BD Biosciences), Ly6G (clone RB6-8C5, eBioscience; clone 1A8, BD Biosciences; clone 1A8, BioLegend), Ly6C (clone HK1.4, BioLegend), CD43 (clone S7, BD Biosciences), CD11b (clone M1/70, Thermo Fisher Scientific; clone M1/70, BioLegend), CCR2 (clone SA203G11, BioLegend), IgM (clone II/41, Thermo Fisher Scientific; clone eB121-15F9, Thermo Fisher Scientific; clone R6-60.2, BD Biosciences), IgD (clone 11-26c.2a, BD Biosciences), CD25 (clone PC61, BD Biosciences), CD30 (clone CD30.1, BD Biosciences), CD5 (clone 53-7.3, Thermo Fisher Scientific), CD45.1 (clone A20, BioLegend), and CD45.2 (clone 104, BioLegend; clone 104, eBioscience; clone 104, Thermo Fisher Scientific). Flow antibodies against human included CD3 (clone SK7, BioLegend), CD20 (clone 2H7, BD Biosciences), CD19 (clone HIB1g, BD Biosciences), CD27 (clone L128, BD Biosciences), CD45 (clone 2D1, BioLegend), CD16 (clone 3GB, BD Biosciences), CD14 (clone M5E2, BD Biosciences), CD11b (clone M1/70, BioLegend), CD15(clone W6D3, BioLegend), CCR2 (clone 48607, R&D Systems), IgM (clone G20-127, BD Biosciences), CD5 (clone L17F12, BioLegend), and CD43 (clone 1G10, BD Biosciences). Samples were run on a FACScan 4 (BD). Acquired data were analyzed with FlowJo v10.8 (FlowJo, BD).

### B cell isolation and adoptive transfer experiments

From single-cell suspension, B cell isolation was performed using a negative selection B cell isolation kit (Miltenyi Biotec). For adoptive transfer experiments, B cells were isolated from spleens of B6 WT, CCL2^–/–^, CCL7^–/–^, TLR3^–/–^, TLR4^–/–^, TRIF^–/–^, MyD88^–/–^ IgHEL, and β-actin–GFP mice. Twenty-four hours prior to left lung hilar clamping, approximately 10^6^ B cells were injected via the right internal jugular vein.

### mRNA isolation and RT-qPCR

From single-cell suspension made as described above, total RNA was extracted using an RNeasy Micro Kit (Qiagen) and reverse transcribed with iScript Reverse Transcription Supermix (Bio-Rad). The following primers were used: CCL7 forward (GGTGGCAAGAAGTAGGGTGT), CCL7 reverse (TGGTGTCAGCTTGTCAGAGAC), CCL2 forward (GAGAGCAACACAGGTTGGGA), and CCL2 reverse (GGAAGGACTGGGGCTTTTGT). Relative expression of the transcripts was determined according to the ΔΔCt method using S18 as reference for normalization.

### ELISA

Mouse serum anti-collagen V antibodies were measured using a commercially available ELISA kit (MBS9365767, MyBiosource). Briefly, the plate was precoated with recombinant murine partial collagen V, α1. The serum samples were bound to collagen V with 2 hours of incubation at room temperature. Polyclonal goat anti-mouse IgG, IgM-HRP, and TMB substrate were used for detection, as per the manufacturer’s protocol. The reactions were quantified with optical density measurement at 450 nm using a Synergy/HTX Multimode reader (BioTek).

### Histology and immunostaining

Harvested lung tissues were fixed with 4% paraformaldehyde, embedded in paraffin, and were stained with H&E. For IHC, paraffin sections were deparaffinized and rehydrated, followed by antigen retrieval in citrate buffer (pH 6.0, Vector Laboratories). Endogenous peroxidase activity was quenched with 3% H_2_O_2_, and the nonspecific binding was blocked with serum and avidin/biotin blocking kit (SP2001, Vector Laboratories). Slides were incubated with 1:1,000 rat anti-mouse B220 (clone RA3-6B2, eBioscience), 1:1,000 rat anti-mouse Ly6G (clone 1A8, BioLegend), and 1:200 polyclonal rabbit anti-mouse C4d (HP8033, Hycult Biotech) overnight at 4°C. Following primary antibody incubation, biotinylated secondary anti-rat or anti-rabbit antibodies were applied followed by the Vector ABC elite staining kit (PK-6104, PK-6101) according to the manufacturer’s instructions. Finally, slides were incubated with 3,3′-diaminobenzidine substrate for detection and counterstained with hematoxylin for nuclear staining. For immunofluorescence staining, after antigen retrieval and blocking, 1:100 rat anti-mouse Ly6G (clone 1A8, BioLegend) and 1:100 mouse anti-human CD20 (clone L26, eBioscience) primary antibodies were used. Alexa Fluor (AF) 647–labeled goat anti-rat (Invitrogen, 1:200) and AF-594–labeled goat anti-mouse (Invitrogen, 1:1,000) secondary antibodies were used for visualization. Nuclei were visualized with Hoescht 33342 (Invitrogen). Images were acquired on an Olympus BX61 microscope using CellSens Dimension software (v1.18). Quantification was performed with QuPath (v0.4.3).

### scRNA-Seq

#### Preparation of single-cell suspension.

Orthotopic left lung transplantation was performed from a B6 (CD45.2) donor into a B6 (CD45.1) recipient. Grafts were stored for 60 minutes at 4°C with an additional 45 minutes at 28°C prior to transplantation. The left lung was allowed to reperfuse for either 2 hours or 3 days prior to sacrifice and lung harvest. For the naive sample, the left lung was harvested from a naive B6 mouse. Single-cell suspension was prepared as described above. For each sample, cells from 2 mice were pooled and were stained with CD45.2 (clone 104; Biolegend), CD45.1 (clone A20; Biolegend), and DAPI (BD Biosciences, 564907). Flow cytometric analysis and sorting were performed on a BD FACS Melody cell sorter.

#### Library construction and gene sequencing.

Cells were processed and encapsulated with barcoded oligo-dT containing gel beads with the 10X Genomics Chromium controller. Library preparation was performed as per manufacturer recommended protocols at the Genome Technology Access Center at Washington University. Samples were processed using the Chromium Single-cell 3′ Library & Gel Bead Kit (10X Genomics, v3). Libraries were sequenced on the NovaSeq S4 (Illumina), with a target of 50,000 reads per cell and 500 million read pairs per library.

#### scRNA-Seq analysis.

Analysis was performed using RStudio (Posit) and the R Seurat v4.2.0 package. Raw fastq files were aligned to the mouse genome using CellRanger (v6). Quality control filters were applied to only keep cells with nFeature_RNA >200 & nFeature_RNA <6000 & percent.mt <10 & nCount_RNA <25000. Subsequently, Seurat was used for SCTransform normalization (with regression of mitochondrial percentage and nCounts_RNA), principal component analysis, UMAP embedding construction, and clustering. B cells were selected for further analysis using canonical marker genes. B cells were further clustered into cell states, and differential expression analysis was performed using FindAllMarkers to identify transcriptional signatures of each B cell state (Supplemental Table 1). Similarly, FindAllMarkers was also used to calculate differentially expressed genes across the 3 conditions (naive and 2 hours and 3 days after transplant). Genes were deemed significantly different if log_2_-fold change (log_2_FC) > 0.58 and adjusted *P* < 0.05 using Wilcoxon’s rank-sum test in Seurat. Cluster Profiler was used to perform Gene Ontology pathway analysis across the conditions.

### Statistics

Data analysis was performed using Prism 10 (GraphPad). Results are expressed as mean ± SEM, and the *n* values for each data set are provided in the figure legends. Pearson’s correlation was used to evaluate 2 continuous variables. Hypothesis testing was done by 2-tailed Mann-Whitney or paired *t* test for data with 2 groups. For multiple-group comparisons, we tested the data for normality (Shapiro-Wilk) and analyzed by 1-way ANOVA with post hoc Holm-Šídák or by Kruskal-Wallis with post hoc comparisons for data with more than 2 groups. A *P* value of less than 0.05 was considered statistically significant.

### Study approval

All studies were approved by the Animal Studies Committee at Washington University. Animals received humane care in compliance with the *Guide for the Care and Use of Laboratory Animals* (National Academies Press, 2011) and the *Principles of Laboratory Animal Care* formulated by the National Society for Medical Research. The human protocol was approved by the Washington University Institutional Review Board (no. 201012829). All study participants provided informed written consent.

### Data availability

The data generated in this study have been deposited in the GEO database under accession code GSE249242 (https://www.ncbi.nlm.nih.gov/geo/query/acc.cgi?acc=GSE249242). All data supporting the graphs are provided in the Supplemental Supporting Data Values file.

## Author contributions

KF, YZB, DBM, ADFF, YT, YY, ZL, FL, WL, HMS, and RGN conducted experiments. RRH, VP, KJL, AEG, AB, and DK contributed to study design and reviewed and revised the manuscript. KF, YZB, DBM, JMA, ADFF, and RGN analyzed and interpreted the data. KF, YZB, YY, DK, and RGN wrote the manuscript. RGN designed and supervised the study. Co–first author order was determined by the authors themselves.

## Supplementary Material

Supplemental data

Supplemental table 1

Supplemental video 1

Supplemental video 2

Supporting data values

## Version 1 01/23/2024

In-Press Preview

## Version 2 03/15/2024

Electronic publication
