# Supplementary material for: B cells mediate lung ischemia/reperfusion injury by recruiting classical monocytes via synergistic B cell receptor/TLR4 signaling
Source: J Clin Invest. 2024 Jan 23;134(6):e170118. doi: 10.1172/JCI170118 (PMC10940088; doi:10.1172/JCI170118)
Supplement: Supplemental data [file jci-134-170118-s235.pdf]

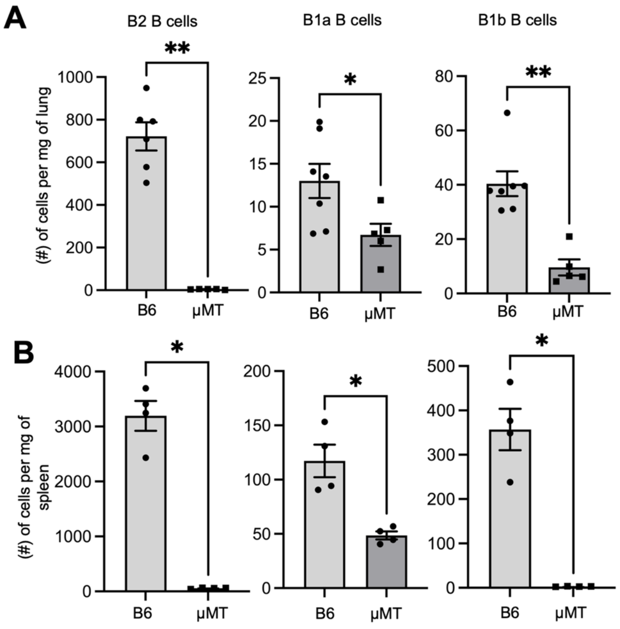

Supplemental Figure 1. ***B1 B cells are present in B cell deficient (μMT) mice, but in significantly smaller numbers than in WT mice.*** Total number of B2 and B1 B cells per mg of **A:** lung and **B:** spleen in B6 WT and μMT mice. Results are presented as mean±SEM, n=7. *p* values calculated by Mann-Whitney test. *$p<0.05$ **$p<0.01$.

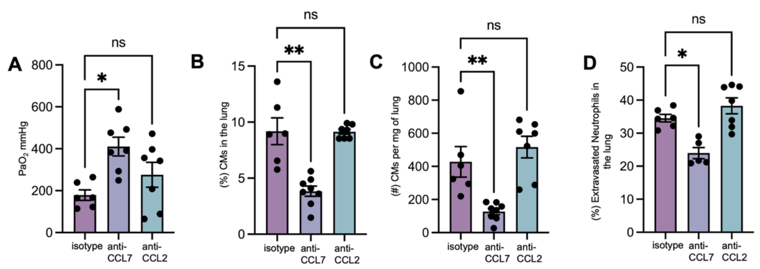

Supplemental Figure 2. ***Treatment with anti-CCL7 improves oxygenation after hilar clamping IRI.*** B6 WT mice were treated with anti-CCL7 antibody, anti-CCL2 antibody or isotype control 24 hours prior to hilar clamping. After one hour of reperfusion, lung function was assessed with ABG, and flow cytometry was used to analyze myeloid populations. **A:** PaO_2 values showed that treatment with anti-CCL7, but not anti-CCL2, antibody improves oxygenation when compared to isotype control. Quantification of **B:** percentage and **C:** number per mg of lung tissue of CMs in the lung. **D:** Percentage of extravasated neutrophils after IRI. Results are presented as mean±SEM, n=4-8. *p* values calculated by Kruskal-Wallis test. *$p<0.05$ **$p<0.01$.

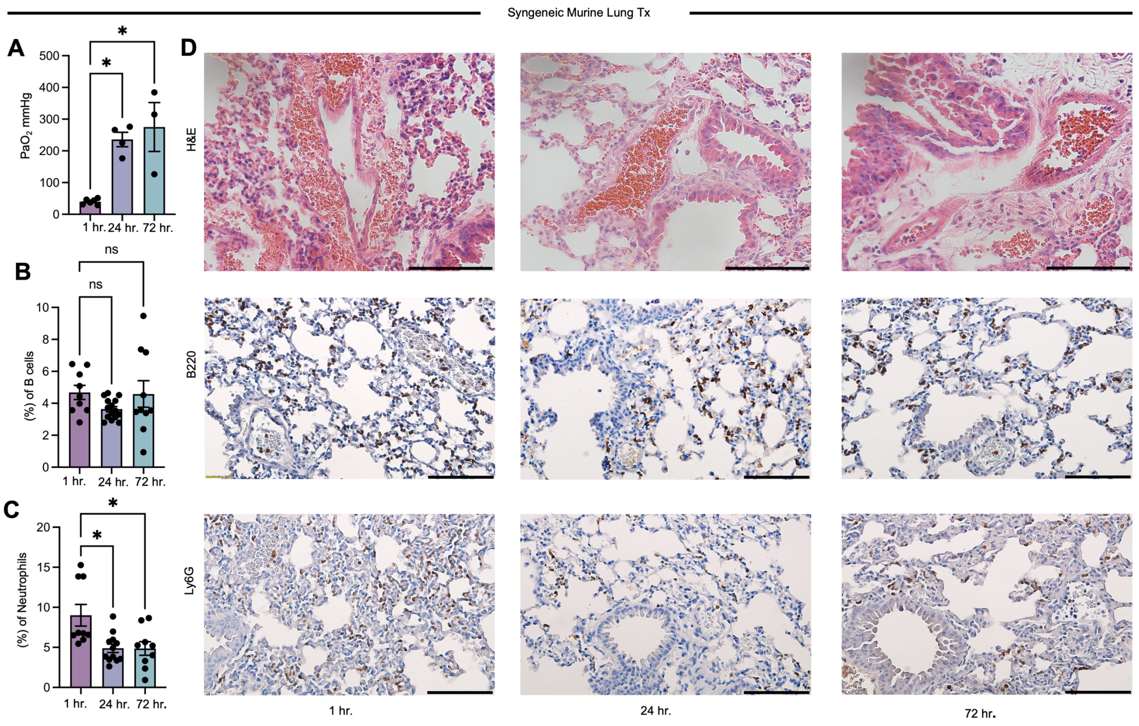

Supplemental Figure 3. ***Improvement of lung function over time after syngeneic lung transplant is associated with a reduction in neutrophil abundance.*** **A:** PaO_2 values after syngeneic lung transplant are significantly improved over a period of 72 hours. Quantification of **B:** B cells and **C:** neutrophils by IHC staining for B220 and Ly6G, respectively, per hpf. **D:** Histological images of lungs after syngeneic lung transplant by H&E staining and IHC staining for B220 and Ly6G. Magnification: 40x, scale bars: 100 µm. Results are presented as mean ± SEM, n=4-8. *p* values calculated by Kruskal-Wallis test. *$p<0.05$. LTx= Lung Transplant.

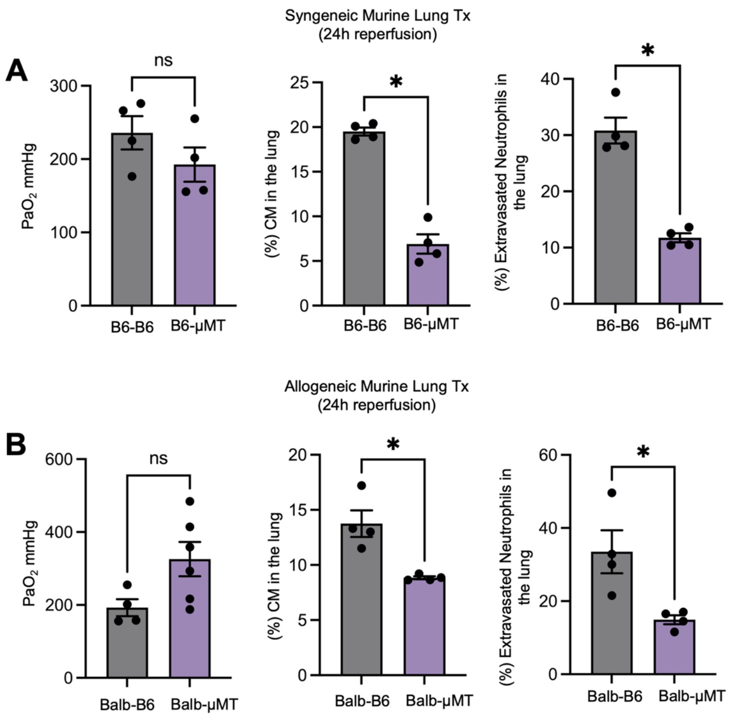

Supplemental Figure 4. ***Absence of recipient B cells is associated with decreased classical monocyte recruitment and decreased neutrophil extravasation at 24 hours post-reperfusion in both syngeneic and allogeneic transplants.*** **A.** PaO_2 values, classical monocyte recruitment to lung grafts, and neutrophil extravasation comparing B6 WT→B6 WT to B6 WT→ B6 μMT syngeneic mouse lung transplants. **B.** PaO_2 values, CM recruitment to lung grafts, and neutrophil extravasation comparing Balb/c→B6 WT to Balb/c→B6 μMT allogeneic mouse lung transplants. Results are presented as mean±SEM, n=4-6. *p* values calculated by Mann-Whitney test. *$p<0.05$. LTx= Lung Transplant.

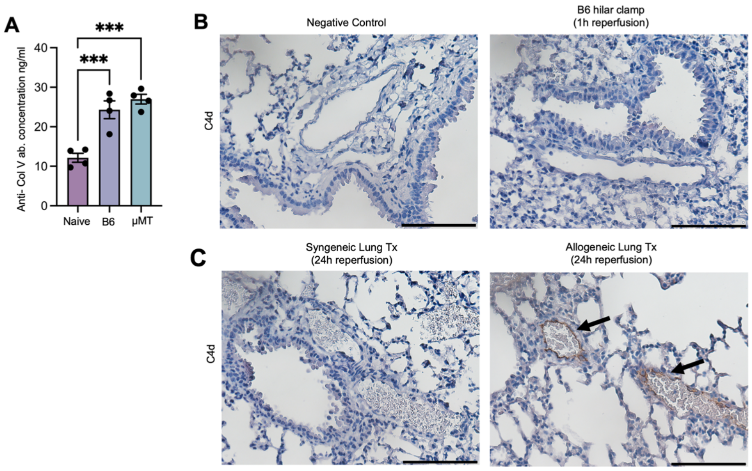

Supplemental Figure 5. ***Lung-infiltrating B cells do not trigger complement deposition following IRI without allogeneic stimuli.*** **A.** Anti-collagen V lung-reactive antibody levels in naïve, B6 WT, and µMT after left lung hilar clamping with 1 hour reperfusion, as measured by ELISA. **B.** IHC staining for complement 4d (C4d) in B6 WT after hilar clamping with 1 hour reperfusion, compared to negative control. **C.** IHC staining for C4d in syngeneic (B6 WT→B6 WT) versus allogeneic (Balb/c→B6 WT) lung transplant 24 hours after reperfusion. Results are presented as mean ± SEM, n=4. *p* values calculated by 1-way ANOVA test. ***$p<0.001$. LTx = Lung Transplant

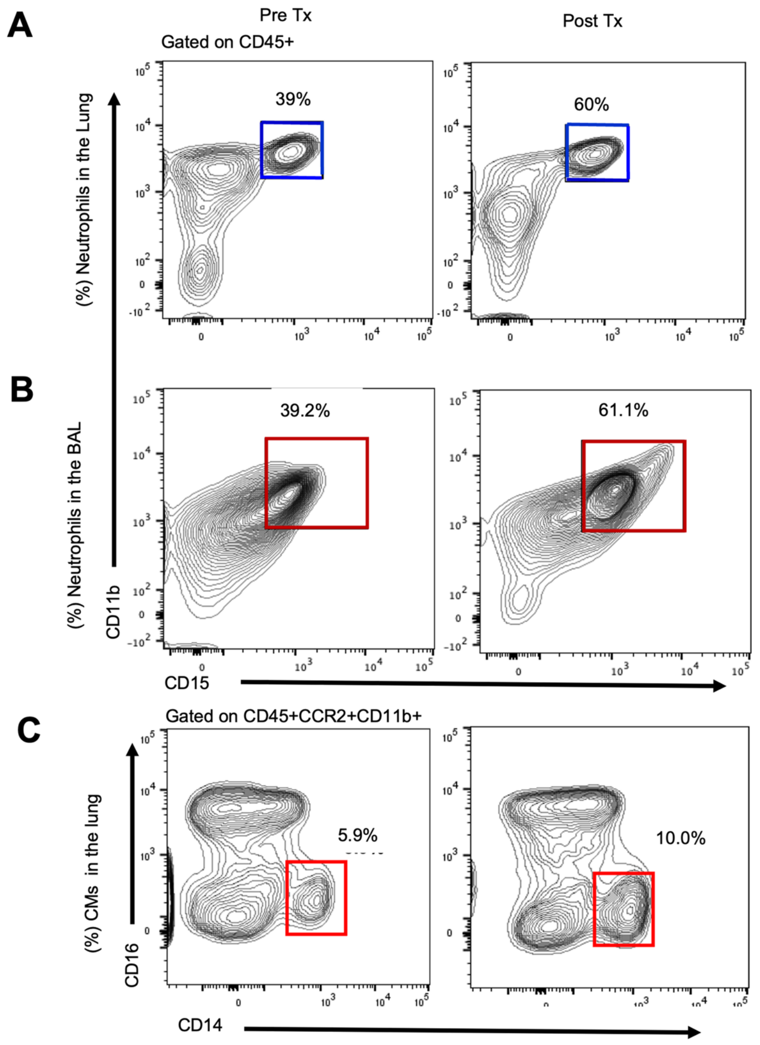

Supplemental Figure 6. Increased recruitment of *neutrophils and classical monocytes to the lung after human lung transplantation.* **A:** Representative flow cytometry contour plots showing percentage of neutrophils ($CD45^{+}CD11b^{+}CD15^{+}$) recruited to the lung and **B:** percentage of neutrophils in BAL (extravasated neutrophils) before and two hours after reperfusion. **C:** Representative flow cytometry contour plots of CMs ($CD45^{+}CCR2^{+}CD11b^{+}CD14^{++}CD16^{-}$) in the lung graft before and two hours after reperfusion. All plots represent n=4-9. BAL= Bronchoalveolar lavage.

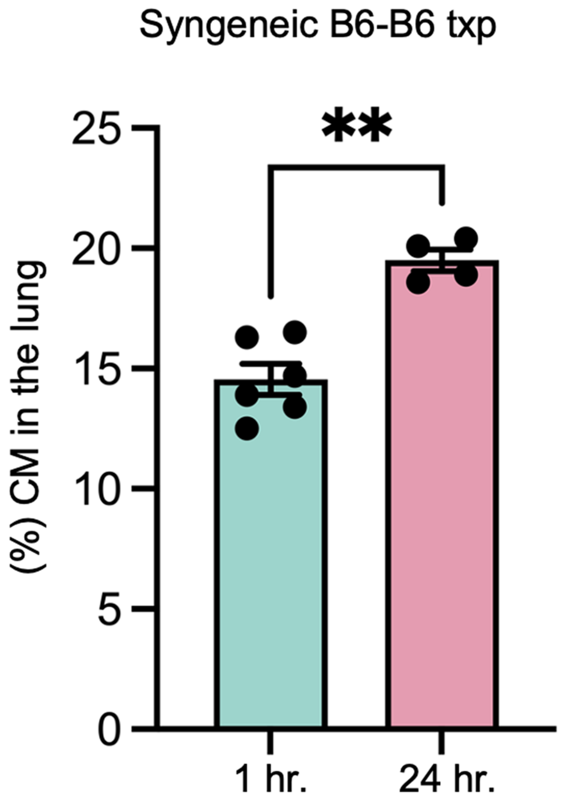

Supplemental Figure 7. ***Classical monocyte recruitment to the lung continues until at least 24 hours post-reperfusion following syngeneic transplant.*** Quantification of the percentage of CMs in the lung after syngeneic B6→B6 transplant after 1 versus 24 hours of reperfusion. Results are presented as mean±SEM, n=4-6. *p* values calculated by Mann-Whitney test. **p<0.01. Txp= Transplant.

Supplemental Table 1. Differentially expressed genes between recipient B cell clusters 0-7, ordered by cluster number and in descending order of average log2FC. *p* values calculated by Wilcoxon-Rank Sum test. *p* values less than 0.05 are considered significant.

Supplemental Video 1. Time-lapse intravital two-photon imaging demonstrating recipient-derived B cells (green) entering vasculature one hour after reperfusion of syngeneic lung transplant. Dextran-rhodamine B was used to label blood vessels (red).

Supplemental Video 2. Time-lapse intravital two-photon imaging demonstrating recipient-derived B cells (green) extravasating into the lung parenchyma (highlighted by yellow line) one hour after reperfusion of syngeneic lung transplant. Dextran-rhodamine B was used to label blood vessels (red).
